# Selective transition-metal-free vicinal *cis*-dihydroxylation of saturated hydrocarbons†
†Electronic supplementary information (ESI) available. See DOI: 10.1039/c6sc03055f



**DOI:** 10.1039/c6sc03055f

**Published:** 2016-08-22

**Authors:** Luis Bering, Andrey P. Antonchick

**Affiliations:** a Max-Planck-Institut für molekulare Physiologie , Abteilung Chemische Biologie , Otto-Hahn-Straße 11 , 44227 Dortmund , Germany . Email: Andrey.Antonchick@mpi-dortmund.mpg.de; b Technische Universität Dortmund , Otto-Hahn-Straße 4a , 44227 Dortmund , Germany

## Abstract

A transition-metal-free *cis*-dihydroxylation of saturated hydrocarbons under ambient reaction conditions has been developed. The described approach allows a direct and selective synthesis of vicinal diols. The new reaction thereby proceeds *via* radical iodination and a sequence of oxidation steps. A broad scope of one-pot dual C(sp^3^)–H bond functionalization for the selective synthesis of vicinal *syn*-diols was demonstrated.

## Abstract 2

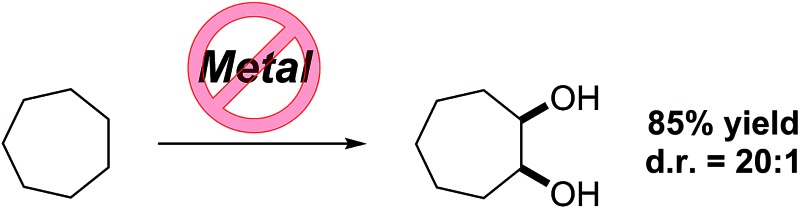
A selective vicinal dihydroxylation of alkanes by dual C(sp^3^)–H bond functionalization under transition-metal-free reaction conditions has been developed.

## Introduction

**Scheme 1:**
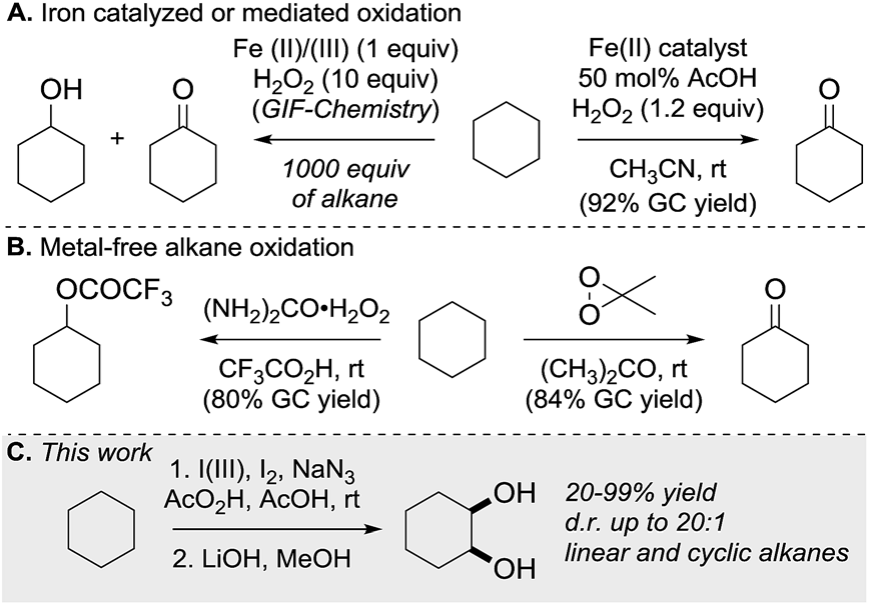
Direct C(sp^3^)–H bond oxidation of alkanes.

C–H bond functionalization of aliphatic hydrocarbons represents a long-standing goal in organic chemistry.^1^ The selective functionalization of the chemically inert and ubiquitous C(sp^3^)–H bond is a great challenge. The participation of hydrocarbons in chemical reactions often requires high temperatures at the expense of the controllability and economy of product formation.^2^ As well as aliphatic C–H bond halogenation, alkylation, aminations, dehydrogenation and borylation, direct C–H bond hydroxylation has gained significant interest.^3^ Alcohols and polyols are important synthetic precursors, structural motifs in natural products and bioactive molecules and are employed in numerous industrial processes.^4^ To date a variety of metal-based, cytochrome P450 inspired and metal-free methods for the hydroxylation of unactivated C–H bonds exist.^5^ Since 1983, Barton and co-workers pioneered the iron-mediated oxidation of alkanes under mild conditions (GIF chemistry) and more recently White *et al.* developed an iron-based catalyst that efficiently oxidizes one equivalent of cyclohexane to the corresponding ketone (Scheme 1A).^6^ In the absence of metal catalyst, the oxidation of hydrocarbons was realized using oxidants in the presence of strong acids or dimethyldioxirane and its analogues (Scheme 1B).^7^ Overoxidation, dehydration and low selectivity are common outcomes of the described methods. 1,2-Diols have high importance in organic synthesis and have found wide application. State of the art methods for diol synthesis are predominately based on the dihydroxylation of alkenes in a metal-catalyzed or metal-free manner.^8^ Despite the progress towards efficient and selective aliphatic C–H bond mono-functionalization, practical methods enabling the direct vicinal dihydroxylation of hydrocarbons are not known. Herein we report the first selective vicinal *cis*-dihydroxylation of saturated hydrocarbons (Scheme 1C). The developed method allows the synthesis of *cis*-diols under transition-metal-free and ambient reaction conditions.

## Results and discussion

**Scheme 2:**
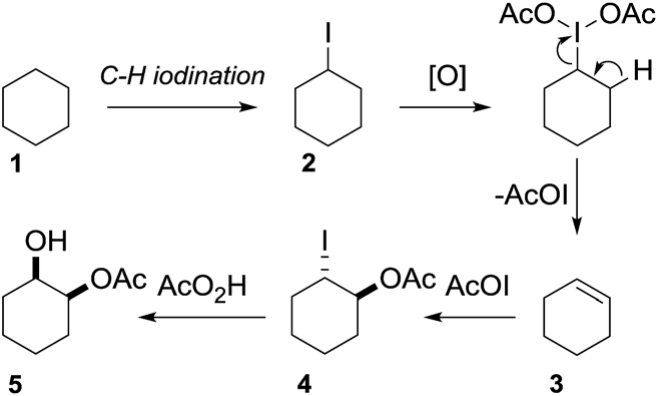
Proposal for the vicinal dihydroxylation.

In 2002 Barluenga and co-workers reported the metal-free functionalization of cyclohexane (**1**) using phenyliodine diacetate (PIDA), I_2_ and *t*BuOH (Scheme 2).^9^ The reaction proceeds *via* the formation of iodocyclohexane (**2**), further oxidation and the *trans*-addition of acetylhypoiodide (AcOI) to give 2-iodohexyl acetate (**4**). Critical for the transformation is the generation of an alkene intermediate through the elimination of AcOI. The same course of reaction was proposed by Sudalai *et al.* ^10^ Nevertheless, a high excess of alkane (200 equiv.) and elevated temperatures were required in both cases to achieve products. In the course of our studies exploring radical reactions and the functionalization of simple alkanes mediated by hypervalent iodine(iii) reagents, we considered the transient formation of iodoalkanes and further oxidation in a cascade reaction fashion as a promising approach for the synthesis of diols from saturated hydrocarbons.^11^ During our primary screening we found that, beside known hypervalent iodine based methods, the PIDA-I_2_-NaN_3_ system enabled radical iodination.^9,10,12^ Furthermore, we hypothesized that by combining radical iodination with an excess of oxidant, such as peracetic acid, epoxidation of the generated alkene would take place and subsequent ring opening would lead to the formation of *trans*-2-hydroxycyclohexyl acetate. However, during the extensive screening, we found that the *trans*-addition of AcOI cannot be bypassed, but it is possible to accomplish an additional oxidation step of 2-iodocyclohexyl acetate (**4**) (Scheme 2). The formation of mono-acetylated diol **5** proceeds in this case *via* the oxidative nucleophilic substitution of iodine.^13^ We were pleased to find that the iodination and the sequence of oxidations could be performed as a one-pot reaction. Through hydrolysis of the crude product, *cis*-cyclohexane-1,2-diol (**6**) was obtained in 72% yield with a relative diastereoselectivity of 5.3 : 1 (Table 1, entry 1). The systematic optimization was started by screening different solvents, but no improvement was found (Table 1, entries 2–6 and see the ESI†). The usage of AcOH was convenient, since AcO_2_H is a solution in AcOH. Among all of the tested oxidants, solely the use of peracetic acid yielded product **6** and an optimum was found when using 12.5 equiv. of oxidant (Table 1, entries 7–10 and see the ESI†). KI and NIS were examined as alternative iodine sources, but they yielded only traces of product (see the ESI†). Significant improvements were achieved during the screening of aryl iodides (Table 1, entries 11–16 and see the ESI†). 4-Iodotoluene yielded diol **6** in 99% yield with respect to the aryl iodide and slightly improved the diastereoselectivity (up to 6.5 : 1). Further screening of the additives revealed the substoichiometric use of iodine to be most advantageous (see the ESI†). High atom efficiency is a typical feature of iodination *via* radical activation.^9,12^ In contrast, the increased loading of I_2_ lowered product formation. We assume that unwanted radical scavenging disturbed the propagation of the reaction. Next, different azides were tested, but the initially used 2.5 equiv. of NaN_3_ yielded the highest product formation (see the ESI†). Finally, we stressed our novel system by reducing the amount of alkane as much as possible. Unchanged product formation was still observed when 8.5 equiv. of cyclohexane was added to the reaction (Table 1, entries 17–19 and see the ESI†). Nevertheless, the application of 8.5 equiv. of cyclohexane is one of the lowest reported loadings for the direct hydroxylation of simple alkanes. The obtained diol **6** is a valuable precursor for the production of adipic acid which is one of the most important chemical intermediates in industry.^14^ Having the optimized conditions in hand, we started to explore the scope of this novel method for transition-metal-free dihydroxylation alkanes by testing cyclic, linear and branched saturated hydrocarbons at a higher scale (Table 2). Gratifyingly, by varying the ring size to 5, 7 and 8 carbons the desired products were obtained in good to moderate yields. Comparable to cyclohexane-1,2-diol (**6**), *cis*-cyclopentane-1,2-diol (**8**) was formed in 86% yield and with a d.r. of 6.3 : 1. Significant improvement of diastereoselectivity was observed with the increasing ring size of the hydrocarbons. Only trace amounts of the *trans*-diastereomer of diol **10** were detected and cyclooctane (**11**) yielded exclusively the *cis*-isomer. Interestingly, the reaction conditions developed by Barluenga and co-workers were used to convert cycloheptane (**9**) into 2-iodo-1-methylcyclohexyl acetate, while our developed reaction conditions smoothly formed the desired diol without ring contraction.^9,12^ Furthermore, the results suggest that the relative stereochemistry is influenced by the neighboring group effect of the acetate group during the oxidative displacement of iodine.^13*c*^ We assume that this effect becomes less dominant with increasing ring size. Next, tertiary (3°) carbon containing alkanes were tested. Although iodination predominantly occurred on the 3° position, minor functionalization of secondary (2°) positions occurred as well. The straightforward benzoylation of diols allowed the isolation of **14a** and **b** and **16a** and **b** in good yields (Table 2, entry 5 and 6) and revealed, that the functionalization of the 3° position proceeded with a high regioselectivity. A remarkable improvement regarding the selectivity of the radical iodination was observed when using 1,4-dimethylcyclohexane (**17**). Offering two 3° carbon atoms for iodination reduced the probability of the functionalization of the 2° C–H position and only traces of product **18b** were formed, while **18a** was isolated in good yields and excellent regioselectivity (Table 2, entry 7). The dihydroxylation of linear alkanes was realized in good yields as well (Table 2, entries 8 and 9). The benzoylation of 2° hydroxyl groups was applied to facilitate the handling of volatile compounds. Major products were identified as a result of the iodination of 2° carbon position and further transformation to give **20a** and **b** and **22a** and **b**. Although oxidation predominantly occurred at 2° carbon atoms, minor product formation as a result of 1° C–H bond iodination or elimination to a terminal double bond was observed (**20c** and **22c**). Finally, branched alkanes containing 3° carbon atoms were tested in the reaction. 3-Methylpentane (**23**) yielded product **24** in 55% yield and demonstrated regioselectivity in a comparable manner to the previous examples. Elimination of oxidized iodine to the thermodynamically more stable double bond had a strong influence on the distribution of regioisomers. Interestingly, due to steric hindrance, product **26** revealed an opposite ratio of regioisomer formation, since dihydroxylation of a terminal double bond took preferential place. Although in the absence of 3° positions C–H functionalization *via* iodination resulted in the formation of regioisomers, it should be noted that among all possible oxidation products exclusively vicinal oxidation was observed and moreover overoxidation to ketones was not detected. Attempts to use aliphatic ethers, esters or carboxylic acids remained unsuccessful under the developed conditions.

**Table 1:** Optimization of reaction conditions^*a*^

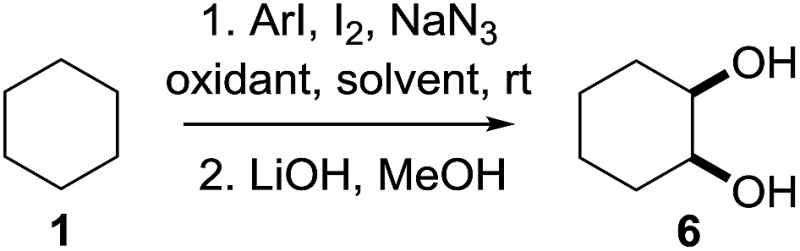					
Entry	Oxidant (equiv.)	ArI or ArI(iii)	Solvent	Yield^*b*^ (%)	d.r.^*c*^
1	AcO_2_H (16.5)	PIDA	AcOH	72	5.3 : 1
2	AcO_2_H (16.5)	PIDA	HCO_2_H	32	7 : 1
3	AcO_2_H (16.5)	PIDA	HFIP	18	1.5 : 1
4	AcO_2_H (16.5)	PIDA	CH_3_CN	16	1.5 : 1
5	AcO_2_H (16.5)	PIDA	CH_2_Cl_2_	20	1.5 : 1
6	AcO_2_H (16.5)	PIDA	w/o	17	4.3 : 1
7	H_2_O_2_ (16.5)	PIDA	AcOH	n.d.	—
8	TBHP (16.5)	PIDA	AcOH	n.d.	—
9	*m*CPBA (16.5)	PIDA	AcOH	n.d.	—
10	Na_2_S_2_O_8_ (16.5)	PIDA	AcOH	Traces	—
11	AcO_2_H (12.5)	PIFA	AcOH	53	5 : 1
12	AcO_2_H (12.5)	DIBA	AcOH	97	5 : 1
13	AcO_2_H (12.5)	4-MeC_6_H_4_I	AcOH	99	6 : 1
14	AcO_2_H (12.5)	4-FC_6_H_4_I	AcOH	79	6.3 : 1
15	AcO_2_H (12.5)	2-MeC_6_H_4_I	AcOH	53	5 : 1
16	AcO_2_H (12.5)	IBA	AcOH	8	10 : 1
17^*d*^	AcO_2_H (12.5)	4-MeC_6_H_4_I	AcOH	99	6.5 : 1
18^*e*^	AcO_2_H (12.5)	4-MeC_6_H_4_I	AcOH	86	6 : 1
19^*f*^	AcO_2_H (12.5)	4-MeC_6_H_4_I	AcOH	8	n.c.

^*a*^Reaction conditions: (1) ArI or ArI(iii) (0.6 mmol, 1 equiv.), oxidant (see table), cyclohexane (12.5 equiv.), I_2_ (0.8 equiv.), NaN_3_ (2.5 equiv.), AcOH (0.1 M), rt, 24 h; (2) LiOH (2 equiv.), MeOH (0.2 M), rt.

^*b*^Yields are given for isolated products after column chromatography. Calculated based on ArI or ArI(iii).

^*c*^Diastereomeric ratio (d.r.) according to ^1^H-NMR.

^*d*^8.5 equiv.

^*e*^6.25 equiv. of cyclohexane.

^*f*^1 equiv. of cyclohexane. Abbreviations: n.d. = not detected, w/o = without, HFIP = hexafluoroisopropanol, PIFA = phenyliodine bis(trifluoroacetate), DIBA = 2,2′-diiodo-4,4′,6,6′-tetra-methylbiphenyl, IBA = 2-iodobenzoic acid, n.c. = not calculated.

**Table 2:** Scope of the vicinal dual C(sp^3^)–H bond hydroxylation of various saturated hydrocarbons^*a*^

Entry	Substrate	Products^*b*^	r.r.^*c*^	Yield^*d*^ (%)
1	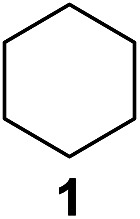	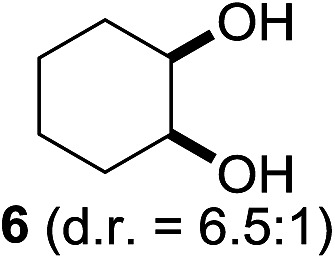	—	99
2	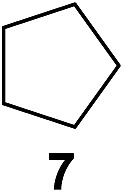	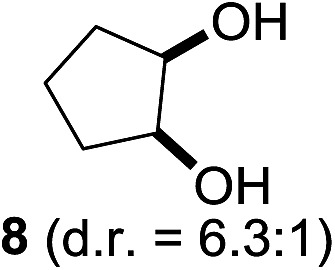	—	86
3	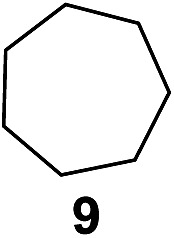	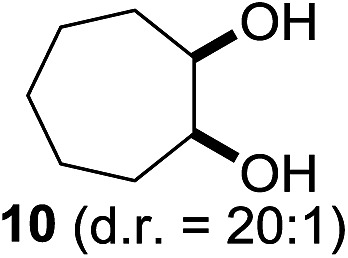	—	85
4	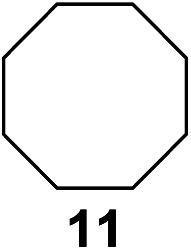	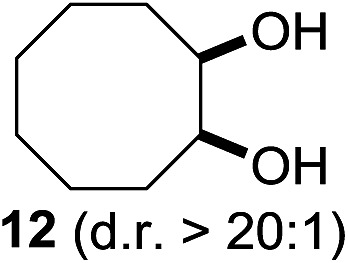	—	37
5^*e*^ ^,^ ^*f*^ ^,^ ^*g*^	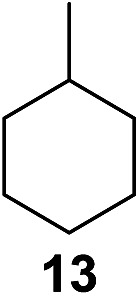	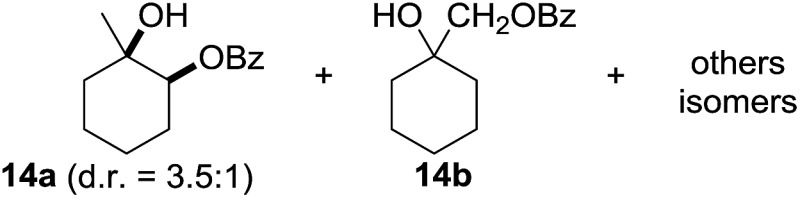	21 : 1 : 7	90 (68^*h*^)
6^*e*^ ^,^ ^*f*^ ^,^ ^*g*^	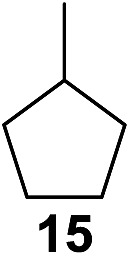	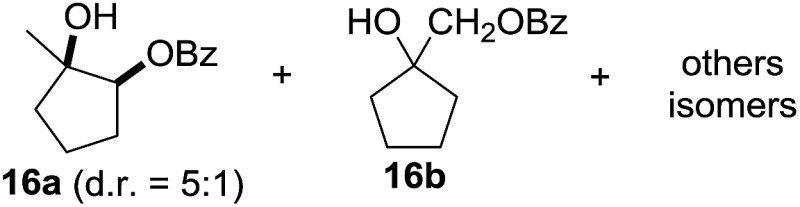	18.5 : 1 : 5	91 (73^*i*^)
7^*e*^ ^,^ ^*f*^ ^,^ ^*g*^	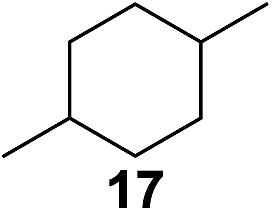	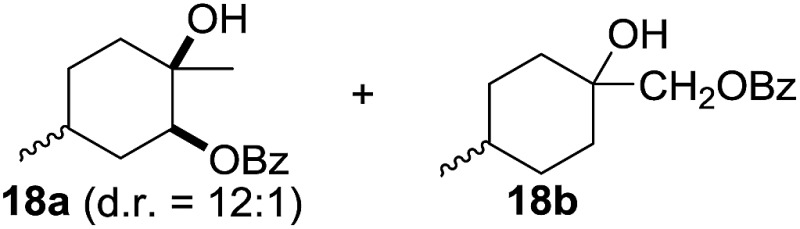	>20 : 1	86
8^*e*^ ^,^ ^*g*^	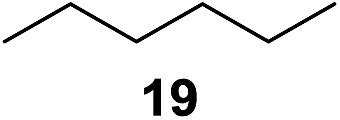	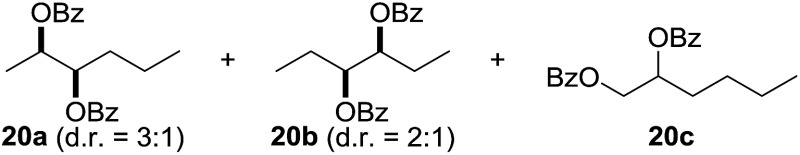	6.5 : 1.5 : 1	68
9^*e*^ ^,^ ^*g*^	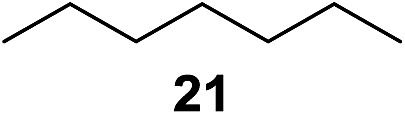	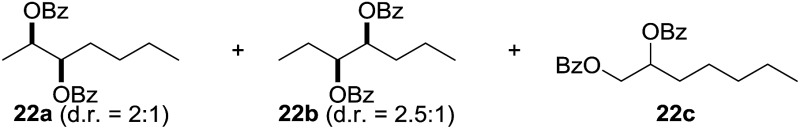	5.5 : 2 : 1	73
10^*e*^ ^,^ ^*f*^ ^,^ ^*g*^	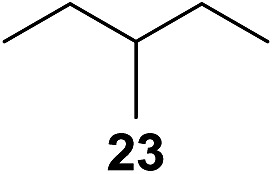	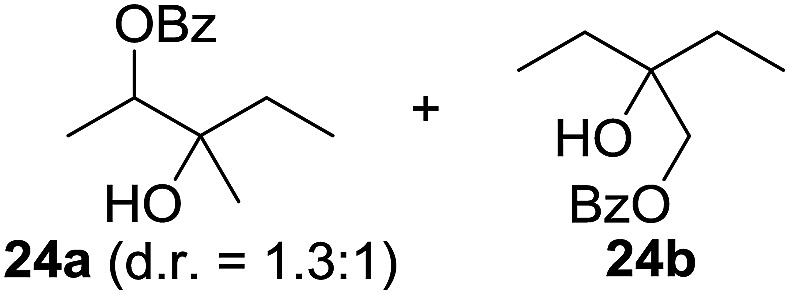	7.7 : 1	55
11^*e*^ ^,^ ^*f*^ ^,^ ^*g*^	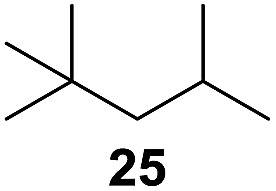	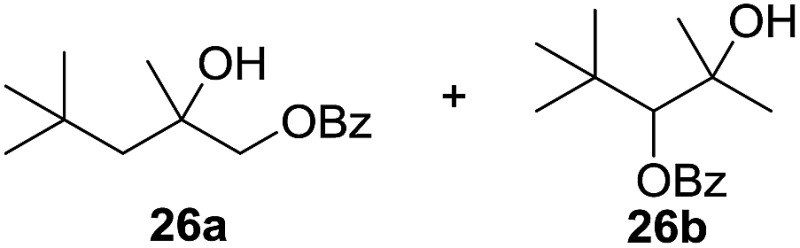	8 : 1	20

^*a*^Reaction conditions: (1) alkane (8.5 equiv.), 4-MeC_6_H_4_I (0.3 mmol, 1 equiv.), I_2_ (0.8 equiv.), NaN_3_ (2.5 equiv.) and AcO_2_H (12.5 equiv.) in AcOH (0.1 M), rt, 24 h; (2) LiOH (2 equiv.), MeOH (0.1 M), rt.

^*b*^Diastereomeric ratio (d.r.) according to ^1^H-NMR. Major isomer is shown.

^*c*^Regioisomeric ratio (r.r.) according to ^1^H-NMR.

^*d*^Yields are given for isolated products. Calculated based on 4-MeC_6_H_4_I.

^*e*^Scaled to 0.9 mmol of 4-MeC_6_H_4_I.

^*f*^3.5 equiv. of NaN_3_.

^*g*^BzCl (2.5 equiv.) and DMAP (5 mol%) in DCM/Py (0.45 M).

^*h*^Yield of isolated regioisomers **14a** and **14b**.

^*i*^Yield of isolated regioisomers **16a** and **16b**.

**Scheme 3:**
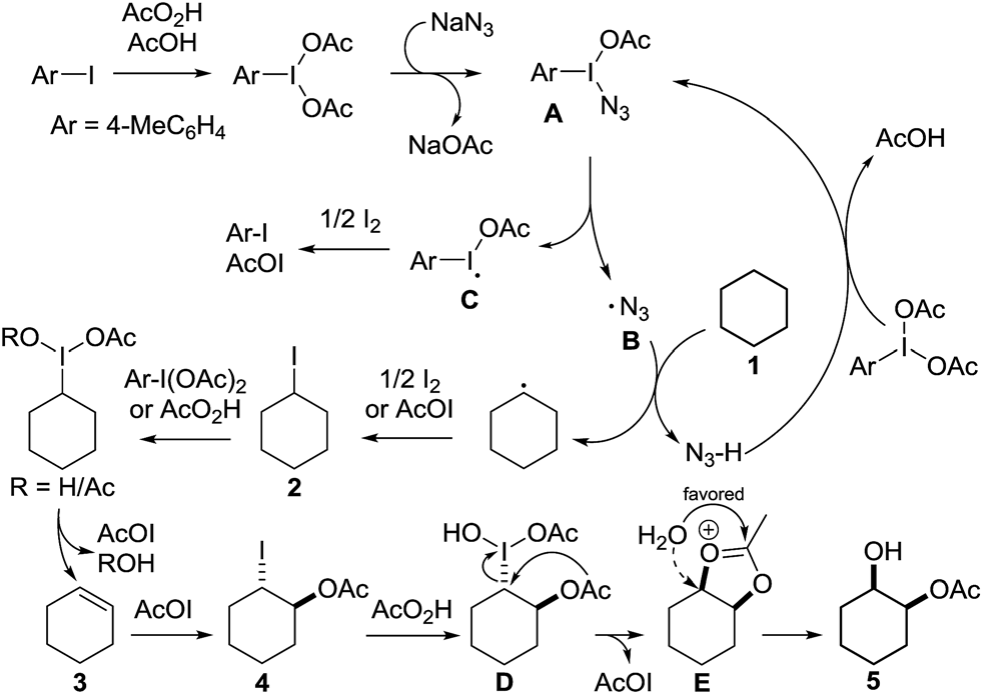
Plausible reaction mechanism.

Having established the scope of the vicinal dihydroxylation using saturated hydrocarbons, the reaction mechanism was studied. By omitting the aryl iodide, iodine or sodium azide no product formation took place. The application of iodocyclohexane instead of cyclohexane smoothly yielded the expected product which was isolated in 98% yield (see ESI†). Additionally, we were able to study the reaction profile by means of GC-MS. The monitoring of the important intermediates **2** and **4** and product **5** formation was possible (see the ESI† and Scheme 3). A significant kinetic deuterium isotope effect was observed (KIE = 7.4), when using *d*_12_-cyclohexane and **1** (see the ESI†). Consequently, the abstraction of hydrogen from cyclohexane is the rate-limiting step. A proposed reaction mechanism is outlined in Scheme 3. Initially 4-iodotoluene is oxidized by peracetic acid in the presence of acetic acid. Ligand exchange with NaN_3_ leads to intermediate **A**, which undergoes thermolysis at ambient temperature to give an azide radical (**B**) and an iodine centred radical (**C**).^11*a*^ **C** is scavenged by iodine whereupon AcOI and 4-iodotoluene are formed. The azide radical reacts with **1** providing a cyclohexyl radical. This transformation is the rate limiting step of the developed cascade reaction. In the following step, the cyclohexyl radical is trapped by iodine or AcOI to provide iodocyclohexane (**2**). The principal difference compared to the reaction conditions developed by Barluenga and co-workers^9^ is the application of sodium azide instead of *t*BuOH.

The use of azide radicals allows a dramatically reduced loading of alkanes and enables iodination at ambient temperature, which makes the formation of iodoalkanes more efficient. The formation of IN_3_ as an intermediate^10^ was excluded, since the formation of 1-azido-2-iodocyclohexane or 2-azidocyclohexyl acetate was not observed under the developed reaction conditions. In control experiments iodocyclohexane (**2**) was converted to product **5** under the developed reaction conditions (see the ESI†). Next, iodocyclohexane (**2**) is oxidized and the reductive elimination of AcOI leads to cyclohexene (**3**). The subsequent *trans*-addition of AcOI to cyclohexene (**3**) provides **4**. Further oxidation of **4** by peracetic acid and nucleophilic substitution gives product **5**.^13*c*^ The stereochemical outcome can be reasoned through a competition between a Woodward- and Prévost-type reaction.^13c,15^ The Woodward-type reaction was favoured for cyclic alkanes. It is important to note that under the developed reaction conditions epoxidation of cyclohexene by peracetic acid (**3**) does not occur. A control experiment revealed the selective formation of *trans*-cyclohexane-1,2-diol after hydrolysis upon oxidation of cyclohexene (**3**) with peracetic acid (see the ESI†). This finding highlights the importance of AcOI which is formed *in situ*.

**Scheme 4:**
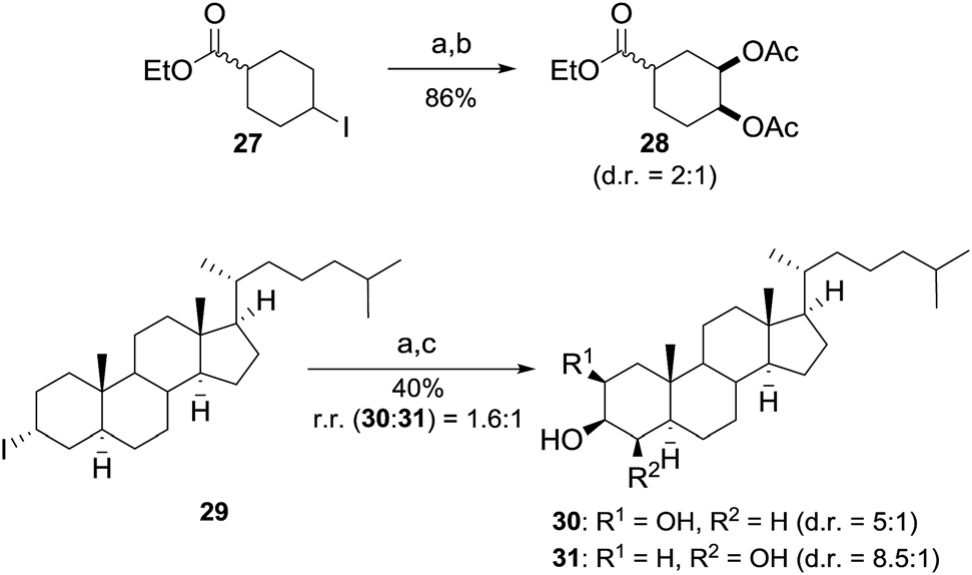
Dihydroxylation of iodoalkanes. Reaction conditions: ^a^ iodoalkane (1 equiv., 0.5 mmol), 4-MeC_6_H_4_I (0.6 equiv.), AcO_2_H (7.5 equiv.) in AcOH (0.3 M), rt, 24 h; ^b^ Ac_2_O (3 equiv.), DMAP (5 mol%) in DCM/Py (0.45 M); ^c^ LiOH (2 equiv.), MeOH (0.1 M), rt.

The application of C–H bond functionalization for the selective derivatization of complex molecules has gained great interest and offers unique advantages in terms of efficiency and atom economy.^16^ However, the required excess of starting material in this protocol negotiates these advantages. Based on the results obtained during the studies on the reaction mechanism, we envisioned that the use of iodoalkanes offers the opportunity to selectively introduce vicinal diols into more complex substrates by mimicking the developed reaction conditions. In this process radical iodination is excluded and the iodine atom in alkyl iodide plays the role of a traceless directing group for metal-free dihydroxylation. According to our proposal, ester **27** yielded the dioxygenated product **28** in a good yield using 1 equiv. of starting material (Scheme 4). Furthermore, the functionalization of complex molecules was tackled by using cholestane derivative **29**. The dihydroxylated products were isolated with good *cis* selectivity after hydrolysis in 40% yield (Scheme 4). It is notable that **29** contains 7 weak tertiary C–H bonds, which were untouched under the applied reaction conditions. It must be noted, that common reaction routes include multiple reaction steps and the use of expensive and toxic osmium catalysts or are mediated by metal reagents.^17^

## Conclusions

We have developed the first efficient and scalable method for the transition-metal-free double C(sp^3^)–H bond functionalization of saturated hydrocarbons. Cyclic, linear and branched alkanes were converted selectively to vicinal diols under ambient reaction conditions. Furthermore, we demonstrated for the first time the use of the iodine atom from alkyl iodides as a traceless directing group for metal-free dihydroxylation. Our discovery is grounded in an unprecedented cascade reaction.

## Experimental

Full synthetic characterization, general experimental procedures and detailed optimization and mechanistic studies are provided in the ESI.†
