# Supplementary material for: Selective transition-metal-free vicinal cis-dihydroxylation of saturated hydrocarbons
Source: Chem Sci. 2016 Aug 22;8(1):452–7. doi: 10.1039/c6sc03055f (PMC5298187; doi:10.1039/c6sc03055f)
Supplement: Supplementary file 1 [file SC-008-C6SC03055F-s001.pdf]

Electronic Supplementary Material (ESI) for Chemical Science.
This journal is © The Royal Society of Chemistry 2016

Selective Transition-Metal-Free Vicinal *cis*-Dihydroxylation of Saturated Hydrocarbons

Luis Bering[1,2] and Andrey P. Antonchick[1,2]*

[1]Max-Planck-Institut für molekulare Physiologie, Abteilung Chemische Biologie, Otto-Hahn-Straße 11, 44227 Dortmund, Germany

[2]Technische Universität Dortmund, Fakultät für Chemie und Chemische Biologie, Chemische Biologie, Otto-Hahn-Straße 4a, 44227 Dortmund, Germany

*Email: Andrey.Antonchick@mpi-dortmund.mpg.de

Table of Contents

General 2

Screening of reaction conditions 3

General procedures 8

Physical data of products 10

Control experiments 25

Reaction profile of the 1,2-dihydroxylation of cyclohexane 26

Kinetic isotope effect 27

Copies of ^{1}H and ^{13}C NMR spectra 29

References 47

General

All reactions were carried out under ambient atmosphere at room temperature. Unless otherwise noted, all commercially available compounds were used as provided without any further purification. Solvents for chromatography were laboratory grade. Analytical thin-layer chromatography (TLC) was performed on Merck silica gel aluminum plates with F-254 indicator, visualized by irradiation with UV light. Column chromatography was performed using silica gel Merck 60 (particle size 0.040-0.063 mm). All functionalized alkanes were visualized using *p*-anisaldehyde staining solution (5 mL glacial sulfuric acid, 1.5 mL glacial acetic acid and 3.7 mL *p*-anisaldehyde in 135 mL absolute EtOH).

^{1}H-NMR and ^{13}C-NMR were recorded on a *Bruker DRX400 (400 MHz), Bruker DRX500 (500 MHz)* and *Bruker DRX600 (600 MHz)* spectrometer in $CDCl_3$ as solvent. Spectra were calibrated relative to solvent's residual proton and carbon chemical shift: $CDCl_3$ (δ = 7.26 ppm for ^{1}H-NMR and δ = 77.16 ppm for ^{13}C NMR). Diastereomers are defined in the following manner: $^{\#}$ denotes major- and * minor diastereomer signals. Data are reported in the following order: chemical shift (d) in ppm; multiplicities are indicated br. s. (broadened singlet), s (singlet), d (doublet), t (triplet), q (quartet), m (multiplet); coupling constants (*J*) are given in Hertz (Hz). Low resolution mass spectra (MS-EI, 70 eV) were collected using a GC-MS (GC system 7890A equipped with 5975C detector) produced by Agilent Technologies (column: HP-5MS, 30 m × 0.250 mm × 0.25 μm). High resolution mass spectra were recorded on a *LTQ Orbitrap* mass spectrometer coupled to an *Accela HPLC System* (HPLC column: *Hypersyl GOLD*, 50 mm × 1 mm, 1.9 μm). Fourier transform infrared spectroscopy (FT-IR) spectra were obtained with a *Bruker Tensor 27* spectrometer (ATR, neat) and are reported in terms of frequency of absorption (cm^{-1}). Reported yields of products correspond to isolated compounds after column chromatography.

Safety-concerns: We have not experienced any explosions while conducting our experiments; however, much care should be taken when working with azides as they are potentially explosive compounds. The reaction proceeds exothermically upon addition of azides. Therefore, water bath cooling has to be applied to the reaction, when performing scales above 0.3 mmol.

Screening of reaction conditions

Table S1. Solvent screening[a]

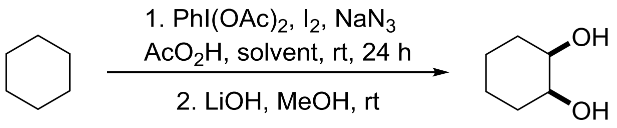

Entry	Solvent	Yield[b] (%)	d.r.[c]
1	AcOH	72	5.3:1
2	formic acid	32	7:1
3	HFIP	18	1.5:1
4	w/o	17	4.3:1
5	CH_3CN	16	1.5:1
6	CH_2Cl_2	20	1.5:1

[a] Reaction conditions: 1. $PhI(OAc)_2$ (0.6 mmol, 1 equiv), AcO_2H (10 mmol, 16.5 equiv), cyclohexane (7.5 mmol, 12.5 equiv), I_2 (0.5 mmol, 0.8 equiv), NaN_3 (1.5 mmol, 2.5 equiv), solvent (0.1 M), rt, 24 h; 2. LiOH (1.25 mmol, 2 equiv), MeOH (0.2 M), rt. [b] Yields are given for isolated products after column chromatography. [c] d.r. according to ^{1}H-NMR.

Table S2. Loading of peracetic acid[a]

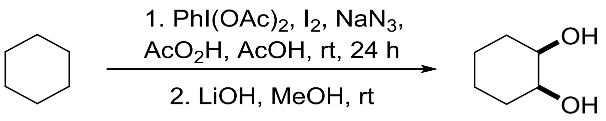

Entry	AcO_2H (equiv)	Yield[b] (%)	d.r.[c]
1	25	11	5.3:1
2	20	53	5.3:1
3	16.5	72	5.3:1
4	12.5	77	5.3:1
5	8	48	5.3:1
6	4	n.d.[d]	-

[a] Reaction conditions: 1. $PhI(OAc)_2$ (0.6 mmol, 1 equiv), AcO_2H (see table), cyclohexane (7.5 mmol, 12.5 equiv), I_2 (0.5 mmol, 0.8 equiv), NaN_3 (1.5 mmol, 2.5 equiv), AcOH (0.1 M), rt, 24 h; 2. LiOH (1.25 mmol, 2 equiv), MeOH (0.2 M), rt. [b] Yields are given for isolated products after column chromatography. [c] d.r. according to ^{1}H-NMR. [d] n.d. = not detected.

Table S3. Oxidant screening[a]

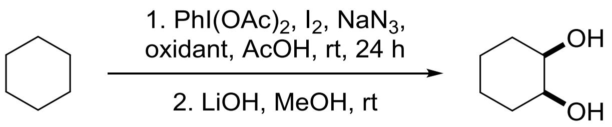

Entry	Oxidant	Yield[b] (%)	d.r.[c]
1	AcO_2H	77	5.1:1
2	H_2O_2 (30% in H_2O)	n.d.[d]	-
3	TBHP (70% in H_2O)	n.d.	-
4	DTBP	n.d.	-
5	perboronate	n.d.	-
6	sodium persulfate	traces	-

[a] Reaction conditions: 1. $PhI(OAc)_2$ (0.6 mmol, 1 equiv), oxidant (7.5 mmol, 12.5 equiv), cyclohexane (7.5 mmol, 12.5 equiv), I_2 (0.5 mmol, 0.8 equiv), NaN_3 (1.5 mmol, 2.5 equiv), AcOH (0.1 M), rt, 24 h; 2. LiOH (1.25 mmol, 2 equiv), MeOH (0.2 M), rt. [b] Yields are given for isolated products after column chromatography. [c] d.r. according to ^{1}H-NMR. [d] n.d. = not detected.

Table S4. Iodine sources[a]

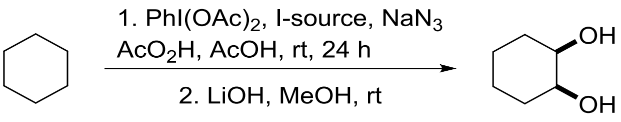

Entry	Iodine source	Yield[b] (%)	d.r.[c]
1	I_2	77	5.3:1
2	KI	5	n.c.[d]
3	NIS	5	n.c.

[a] Reaction conditions: 1. $PhI(OAc)_2$ (0.6 mmol, 1 equiv), AcO_2H (7.5 mmol, 12.5 equiv), cyclohexane (7.5 mmol, 12.5 equiv), iodine source (0.5 mmol, 0.8 equiv), NaN_3 (1.5 mmol, 2.5 equiv), AcOH (0.1 M), rt, 24 h; 2. LiOH (1.25 mmol, 2 equiv), MeOH (0.2 M), rt. [b] Yields are given for isolated products after column chromatography. [c] d.r. according to ^{1}H-NMR. [d] n.c. = not calculated.

Table S5. ArI / ArI(III) screening[a]

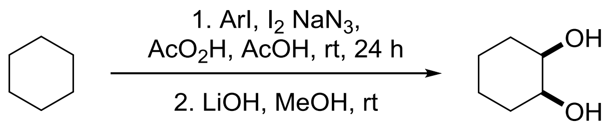

Entry	ArI	Yield[b] (%)	d.r.[c]
1	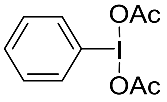	72	5.3:1
2	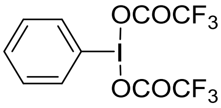	53	5:1
3	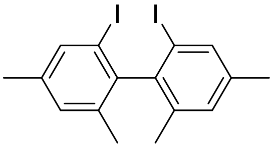	97	5:1
4	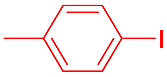	99	6:1
5	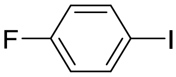	79	6.25:1
6	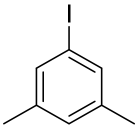	58	4:1
7	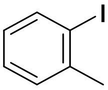	53	5:1
8	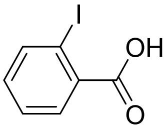	8	10:1
9	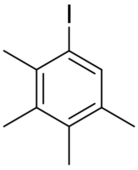	26	5:1
10	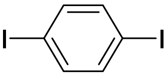	52	5:1
11	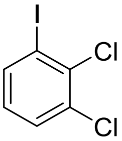	93	4.5:1

[a] Reaction conditions: 1. I-aryl (0.6 mmol, 1 equiv), AcO_2H (7.5 mmol, 12.5 equiv), cyclohexane (7.5 mmol, 12.5 equiv), I_2 (0.5 mmol, 0.8 equiv), NaN_3 (1.5 mmol, 2.5 equiv), AcOH (0.1 M), rt, 24 h; 2. LiOH (1.25 mmol, 2 equiv), MeOH (0.2 M), rt. [b] Yields are given for isolated products after column chromatography. [c] d.r. according to ^{1}H-NMR.

Table S6. Loading of 1-iodo-4-methylbenzene[a]

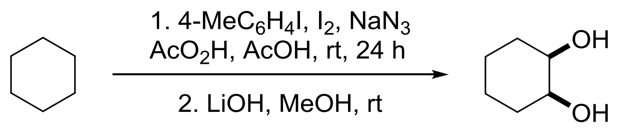

Entry	4-MeC_6H_5I (mmol)	Yield[b] (mmol)	d.r.[c]
1	0.9	0.72	6:1
2	0.6	0.6	6.5:1
3	0.3	0.27	6.1:1
4	0.15	0.14	6.25:1

[a] Reaction conditions: 1. 1-iodo-4-methylbenzene (see table), AcO_2H (7.5 mmol, 12.5 equiv), cyclohexane (7.5 mmol, 12.5 equiv), I_2 (0.5 mmol, 0.8 equiv), NaN_3 (1.5 mmol, 2.5 equiv), AcOH (0.1 M), rt, 24 h; 2. LiOH (1.25 mmol, 2 equiv), MeOH (0.2 M), rt. [b] Yields are given for isolated products after column chromatography. [c] d.r. according to ^{1}H-NMR.

Table S7. Loading of I_2[a]

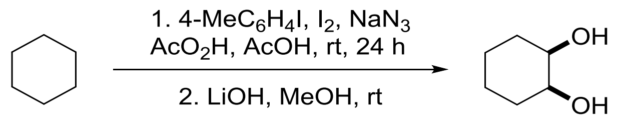

Entry	I_2 (equiv)	Yield[b] (%)	d.r.[c]
1	2	58	6:1
2	1.5	72	6:1
3	1	98	6.5:1
4	0.8	99	6.25:1
5	0.6	89	6.25:1

[a] Reaction conditions: 1. 1-iodo-4-methylbenzene (0.6 mmol, 1 equiv), AcO_2H (7.5 mmol, 12.5 equiv), cyclohexane (7.5 mmol, 12.5 equiv), I_2 (see table), NaN_3 (1.5 mmol, 2.5 equiv), AcOH (0.1 M), rt, 24 h; 2. LiOH (1.25 mmol, 2 equiv), MeOH (0.2 M), rt. [b] Yields are given for isolated products after column chromatography. [c] d.r. according to ^{1}H-NMR.

Table S8. Screening of azides[a]

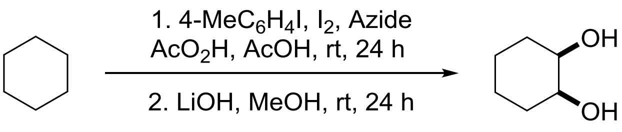

Entry	Azide (equiv)	Yield[b] (%)	d.r.[c]
1	NaN_3 (5)	81	6.3:1
2	NaN_3 (3)	99	6.5:1
3	NaN_3 (2.5)	99	6.5:1
4	NaN_3 (1.5)	95	6:1
5	$TMSN_3$ (2.5)	56	6:1
6	$(PhO)_2PON_3$ (2.5)	16	n.c.[d]

[a] Reaction conditions: 1. 1-iodo-4-methylbenzene (0.6 mmol, 1 equiv), AcO_2H (7.5 mmol, 12.5 equiv), cyclohexane (7.5 mmol, 12.5 equiv), I_2 (0.5 mmol, 0.8 equiv), NaN_3 (see table), AcOH (0.1 M), rt, 24 h; 2. LiOH (1.25 mmol, 2 equiv), MeOH (0.2 M), rt. [b] Yields are given for isolated products after column chromatography. [c] d.r. according to ^{1}H-NMR. [d] n.c. = not calculated.

Table S9. Loading of alkane[a]

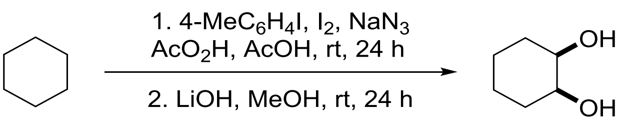

Entry	Alkane (equiv)	Yield[b] (%)	d.r.[c]
1	16.5	98	6:1
2	12.5	99	6.25:1
3	8.5	99	6.5:1
4	6.25	86	6:1
5	1	8	n.c.[d]

[a] Reaction conditions: 1. 1-iodo-4-methylbenzene (0.6 mmol, 1 equiv), AcO_2H (7.5 mmol, 12.5 equiv), cyclohexane (see table), I_2 (0.5 mmol, 0.8 equiv), NaN_3 (1.5 mmol, 2.5 equiv), AcOH (0.1 M), rt, 24 h; 2. LiOH (1.25 mmol, 2 equiv), MeOH (0.2 M), rt. [b] Yields are given for isolated products after column chromatography. [c] d.r. according to ^{1}H-NMR. [d] n.c. = not calculated.

General procedures

Procedure A

To a solution of 4-iodotoluene (65 mg, 0.3 mmol, 1 equiv) in glacial acetic acid (1.4 mL), peracetic acid (704 µL of 39% solution in acetic acid, 3.75 mmol, 12.5 equiv), alkane (2.55 mmol, 8.5 equiv) and iodine (61 mg, 0.24 mmol, 0.8 equiv) were added. When iodine was dissolved (usually within 10 – 15 minutes), NaN_3 (49 mg, 0.75 mmol, 2.5 equiv) was added portionwise over a time period of 15 minutes. The reaction was vigorously stirred for 24 hours at room temperature. After completion, the reaction was diluted with dichloromethane, neutralized with 1 M NaOH solution (25 mL) and washed with saturated Na_2SO_3 solution (10 mL). The aqueous phase was extracted two times with dichloromethane (2x20 mL) and the combined organic layers were dried over Na_2SO_4 and concentrated under reduced pressure. The crude mixture was dissolved in 3 mL MeOH, whereupon LiOH (14.5 mg, 0.6 mmol, 2 equiv) was added and the reaction was stirred at room temperature. After completion, HCl (1.25 M) in MeOH (0.5 mL) was added and MeOH was removed under reduced pressure. Column chromatography purification on silica provided the pure products (eluent: dichloromethane : MeOH).

Procedure B

To a solution of 4-iodotoluene (200 mg, 0.9 mmol, 1 equiv) in glacial acetic acid (4.2 mL), peracetic acid (2.1 mL of 39% solution in acetic acid, 11.25 mmol, 12.5 equiv), alkane (7.65 mmol, 8.5 equiv) and iodine (183 mg, 0.72 mmol, 0.8 equiv) were added. When iodine was dissolved (usually within 10 – 15 minutes), NaN_3 (2.5 equiv or 3.5 equiv; see manuscript, table 2) was added portionwise over a time period of 15 minutes under water bath cooling. Afterwards, the reaction was vigorously stirred for 24 hours at room temperature. After completion, the reaction was diluted with dichloromethane, neutralized with 1 M NaOH solution (75 mL) and washed with saturated Na_2SO_3 solution (30 mL). The aqueous phase was extracted two times with dichloromethane (2x50 mL) and the combined organic layers were dried over Na_2SO_4 and concentrated under reduced pressure. The crude reaction mixture was dissolved in MeOH (9 mL) whereupon LiOH (44 mg, 1.8 mmol, 2 equiv) was added and the reaction was stirred at room temperature. Afterwards, the reaction mixture was quenched with HCl (1.25 M) in MeOH (3 mL) and MeOH was removed under reduced pressure. The crude reaction mixture was purified by column chromatography (eluent: petroleum ether : EtOAc). The obtained diols were dissolved in dichloromethane/pyridine (5 mL, 1:1) and 4-(dimethylamino)-pyridine (5.5 mg, 5 mol%, 0.05 mmol) was added. Benzoyl chloride (261 µL, 2.25 mmol, 2.5 equiv) was added dropwise at 0°C to the reaction mixture and the reaction was stirred for 12 h at room temperature. The excess of benzoyl chloride was quenched by adding 25% NH_4OH solution (1 mL) and the crude reaction was diluted with dichloromethane and washed with saturated $NaHCO_3$ solution (15 mL). The aqueous phase was extracted two times with dichloromethane (2x25 mL) and dried over Na_2SO_4. Column chromatography (eluent: petroleum ether : EtOAc) provided the products**.**

Physical data of products

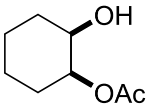

Synthesis of 2-hydroxycyclohexyl acetate (5)

To a solution of $PhI(OAc)_2$ (193 mg, 0.6 mmol, 1 equiv) in glacial acetic acid (2.8 mL) peracetic acid (1.875 mL 39% solution in acetic acid, 10 mmol, 16.5 equiv), cyclohexane (810 µL, 7.5 mmol, 12.5 equiv) and iodine (127 mg, 0.5 mmol, 0.8 equiv) were added. After 15 minutes, NaN_3 (98 mg, 1.5 mmol, 2.5 equiv) was added portion wise over a time period of 15 minutes. Then the reaction was vigorously stirred for 24 hours at room temperature. Afterwards, the reaction was diluted with dichloromethane, slowly neutralized with NaOH solution (1 M, 50 mL) and washed with saturated Na_2SO_3 solution (20 mL). The aqueous phase was extracted two times with dichloromethane (2x40 mL) and the combined organic layers were dried over Na_2SO_4 and concentrated under reduced pressure. Column chromatography provided the pure product (eluent: petroleum ether : EtOAc). The product was obtained as colorless oil (69 mg, 0.43 mmol, 73%). R_f = 0.41 (cyclohexane : EtOAc = 1:1). d.r. = 4.3:1. ^{1}H NMR (500 MHz, $CDCl_3$, $^{\#}$ denotes major-, * minor diastereomer signals) δ 4.91$^{\#}$ (dd, *J* = 8.0, 2.7 Hz, 1H), 4.57* (dd, *J* = 10.1, 4.7 Hz, 1H), 3.88$^{\#}$ (dd, *J* = 6.8, 3.2 Hz, 1H), 3.62 – 3.47* (m, 1H), 2.09$^{\#}$ (s, 2H), 2.08* (s, 3H), 1.84* (m, 4H), 1.81 – 1.72* (m, 4H), 1.62$^{\#}$ (m, 4H), 1.42 – 1.21$^{\#}$ ppm (m, 4H). ^{13}C NMR (126 MHz, $CDCl_3$) δ 170.90, 78.43*, 74.39$^{\#}$, 72.95*, 69.29$^{\#}$, 33.23*, 30.48$^{\#}$, 30.13*, 27.00$^{\#}$, 24.04*, 23.93*, 22.22$^{\#}$, 21.44$^{\#}$, 21.08$^{\#}$ ppm. The spectral data are matching with reported.[4] FT-IR: ν = 3436, 2937, 2863, 2360, 1716, 1237 cm^{-1}. HR-MS: calc. for $[M+H]^+$ $C_8H_{15}O_3$: 159.10157 found 159.10186.

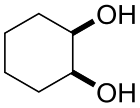

Synthesis of *cis*-cyclohexane-1,2-diol (6)

Prepared according to the general procedure A. The product was obtained as white crystalline solid (34.5 mg, 0.3 mmol, 99%). R_f = 0.34 (cyclohexane : EtOAc = 1:1). d.r. = 6.5:1. ^{1}H NMR (400 MHz, CDCl3) δ 3.78 (dd, *J* = 5.7, 2.5 Hz, 1H), 1.91 (br. s, 2H), 1.82 – 1.69 (m, 2H), 1.69 – 1.49 (m, 4H), 1.40 – 1.21 ppm (m, 2H). ^{13}C NMR (101 MHz, CDCl3) δ 70.77, 30.06, 21.55 ppm. The spectral data are matching with reported.[1] FT-IR: ν = 3392, 3260, 2929, 2851 cm^{-1}. HR-MS: calc. for $[M+H]^+$ $C_6H_{13}O_2$: 117.0966 found 117.09025.

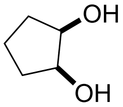

Synthesis of *cis*-cyclopentane-1,2-diol (8)

Prepared according to the general procedure A. The product was obtained as colorless solid (26.5 mg, 0.26 mmol, 86%). R_f = 0.38 (cyclohexane : EtOAc = 1:1) d.r. = 6.3:1. ^{1}H NMR (500 MHz, $CDCl_3$) δ 4.02 (q, *J* = 4.4 Hz, 2H), 2.77 (br. s, 2H), 1.96 – 1.76 (m, 3H), 1.75 – 1.38 ppm (m, 3H). ^{13}C NMR (126 MHz, $CDCl_3$) δ 74.05, 31.19, 19.89 ppm. The spectral data are matching with reported.[2] FT-IR: ν = 3348, 2963 cm^{-1}. HR-MS: calc. for $[M+H]^+$ $C_5H_{11}O_2$: 103.07536 found 103.07518.

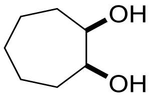

Synthesis of cycloheptane-*cis*-1,2-diol (10)

Prepared according to the general procedure A. The product was obtained as white crystalline solid (33 mg, 0.26 mmol, 85%). R_f = 0.29 (cyclohexane : EtOAc = 1:1). d.r. = 20:1. ^{1}H NMR (400 MHz, $CDCl_3$) δ 3.86 (dd, *J* = 4.0, 1.4 Hz, 2H), 2.18 (br. s, 2H), 1.74 (m, 7H), 1.56 - 1.30 ppm (m, 3H). ^{13}C NMR (101 MHz, $CDCl_3$) δ 73.93, 31.11, 28.00, 22.00 ppm. The spectral data are matching with reported.[2] FT-IR: *ν* = 3333, 2920, 2858 cm^{-1} .HR-MS: calc. for $[M+H]^+$ $C_7H_{15}O_2$: 131.10666 found 131.10593.

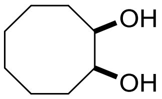

Synthesis of cyclooctane-*cis*-1,2-diol (12)

Prepared according to the general procedure A. The product was obtained as colorless solid (16 mg, 0.11 mmol, 37%). R_f = 0.17 (cyclohexane : EtOAc = 1:1). d.r. > 20:1. ^{1}H NMR (500 MHz, $CDCl_3$) *δ* 3.93 – 3.83 (m, 2H), 2.09 – 1.36 ppm (m, 14H). ^{13}C NMR (126 MHz, $CDCl_3$) *δ* 71.75, 33.47, 30.58, 22.45 ppm. The spectral data are matching with reported.[3] FT-IR: *ν* = 3316, 2924, 2857 cm^{-1}. HR-MS: calc. for $[M+H]^+$ $C_8H_{17}O_2$: 145.12231 found 145.12177.

Dioxygenation of methylcyclohexane (13)

Prepared according to the general procedure B using 3.5 equiv of NaN_3. Column chromatography (eluent: petroleum ether : EtOAc) provided the products in the following order:

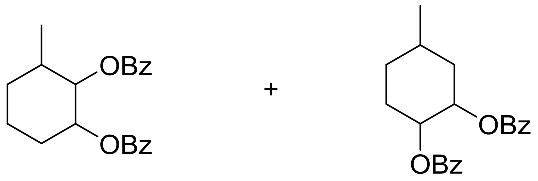

3-Methylcyclohexane-1,2-diyl dibenzoate (14c) **4-Methylcyclohexane-1,2-diyl dibenzoate (14d)**

Inseparable mixture of regio- and diastereomers. The products were obtained as colorless oil (66 mg, 0.2 mmol, 22%). R_f = 0.88 (cyclohexane : EtOAc = 1:1).

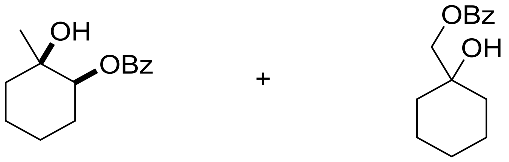

2-Hydroxy-2-methyl-cyclohexyl benzoate (14a) *major* **(1-Hydroxycyclohexyl)-methyl benzoate (14b)** *minor*

d.r. = 3.5:1

Regioisomeric ratio (r.r.) > 20:1

The products were obtained as colorless oil (143 mg, 0.61 mmol, 68%). R_f = 0.69 (cyclohexane : EtOAc = 1:1). r.r. > 20(**14a**):1(**14b**). d.r. = 3.5:1. ^{1}H NMR (400 MHz, $CDCl_3$, $^{\#}$ denotes major-, * minor diastereomer signals) δ 8.13 – 7.94 (m, 2H), 7.55 (t, J = 6.8, 4.1, Hz, 1H), 7.50 – 7.30 (m, 2H), 4.96 (dd, J = 8.9, 4.1 Hz, 1H, 14a*), 4.92 (dd, J = 8.5, 5.8 Hz, 1H, 14a$^{\#}$), 4.23 (s, 2H, 14b), 2.02 (br. s, 1H), 1.90 – 1.79 (m, 2H), 1.79 – 1.54 (m, 2H), 1.44 (m, 2H), 1.31 (s, 3H, 14a*), 1.24 ppm (s, 3H, 14a$^{\#}$). ^{13}C NMR (101 MHz, $CDCl_3$, $^{\#}$ denotes major-, * minor diastereomer signals) δ 166.37 (14a$^{\#}$), 166.01 (14a $^{\#}$), 133.11 (14a $^{\#}$), 133.05 (14a *), 130.58 (14a *), 130.43 (14a $^{\#}$), 129.69 (14a*), 129.67 (14a $^{\#}$), 128.50 (14a $^{\#}$), 128.45 (14a*), 78.87 (14a*), 78.37 (14a$^{\#}$), 77.16 (14a*), 72.05 (14a$^{\#}$), 71.08, 38.13, 37.58, 28.32, 27.32, 27.00, 23.59, 22.89, 22.51, 21.69, 21.29 ppm. FT-IR: ν = 3500, 2935, 2862, 1699, 1268, 1108 cm^{-1}. HR-MS (**14a**): calc. for $[M+H]^+$ $C_{14}H_{19}O_3$: 235.13287 found 235.13245.

Dioxygenation of methylcyclopentane (15)

Prepared according to the general procedure B using 3.5 equiv of NaN_3. Column chromatography (eluent: petroleum ether : EtOAc) provided the products in the following order:

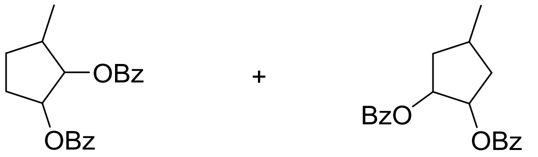

3-Methylcyclopentane-1,2-diyl dibenzoate (16c) **4-Methylcyclopentane-1,2-diyl dibenzoate (16d)**

Inseparable mixture of regio- and diastereomers. The products were obtained as colorless oil (54 mg, 0.17 mmol, 18.5%). R_f = 0.86 (cyclohexane : EtOAc = 1:1).

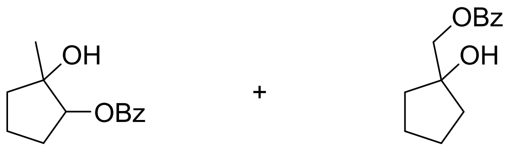

2-Hydroxy-2-methyl-cyclopentyl benzoate (16a) *major* d.r. = 5:1 **(1-Hydroxycyclopentyl)-methyl benzoate (16b)** *minor*

Regioisomeric ratio (r.r.) = 18.5:1

The products were obtained as pale yellow oil (145 mg, 0.66 mmol, 73%). R_f = 0.65 (cyclohexane : EtOAc = 1:1). r.r. = 18.5(**16a**):1(**16b**). d.r. = 5:1. ^{1}H NMR (500 MHz, $CDCl_3$, $^{\#}$ denotes major-, * minor diastereomer signals) δ 8.09 – 7.96 (m, 2H), 7.56 (m, 1H), 7.44 (t, J = 7.7 Hz, 2H), 5.15 – 5.07 (m, 1H, 16a*), 4.99 (t, J = 6.8 Hz, 1H, 16a$^{\#}$), 4.33 (s, 2H, 16b), 2.28 – 1.59 (m, 3H), 1.33 (s, 3H, 16a$^{\#}$), 1.31 ppm (s, 3H, 16a*). ^{13}C NMR (126 MHz, $CDCl_3$, $^{\#}$ denotes major-, * minor diastereomer signals) δ 166.93 (16a*), 166.78 (16b), 166.33 (16a$^{\#}$), 133.23, 130.17, 129.67, 128.52, 84.19 (16a*), 81.26 (16d), 81.00 (16a$^{\#}$), 80.37 (16a*), 78.60 (16a$^{\#}$), 71.37 (16a*), 38.68, 37.38, 36.81 (16a$^{\#}$), 30.46, 28.77 (16a$^{\#}$), 25.42 (16a$^{\#}$), 24.16, 23.25, 20.62, 19.36 (16a$^{\#}$) ppm. FT-IR: ν = 3512, 2965, 1713, 1269, 1109 cm^{-1}. HR-MS (**16a**): calc. for $[M+H]^+$ $C_{13}H_{17}O_3$: 221.11722 found 221.11723.

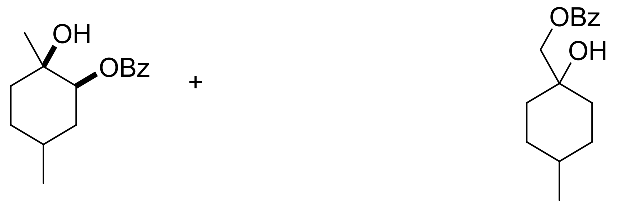

2-hydroxy-2,5-dimethyl cyclohexyl benzoate (18a)
major
d.r. = 12:1

(1-hydroxy-4-methylcyclohexyl) methyl benzoate (18b)
minor

Regioisomeric ratio (r.r.) > 20:1

Dioxygenation of 1,4-dimethylcyclohexane (17)

Prepared according to the general procedure B using 3.5 equiv of NaN_3. The product was obtained as colorless solid (191 mg, 0.77 mmol, 86%). Rf = 0.71 (cyclohexane : EtOAc = 1:1). r.r. > 20(**18a**):1(**18b**). d.r. = 12:1. ^{1}H NMR (500 MHz, $CDCl_3$, # denotes major-, * minor diastereomer signals) δ 8.11 – 7.98 (m, 2H), 7.63 – 7.50 (m, 1H7.45 (t, *J* = 7.7 Hz, 2H), 5.01 (m, 1H, 18a*), 4.92 (dd, *J* = 11.3, 4.6 Hz, 1H, 18a$^{\#}$), 4.19 (s, 2H, 18b), 1.87 – 1.81 (m, 2H), 1.63 – 1.30 (m, 5H), 1.24 (s, 3H, , 18a*), 1.23 (s, 3H, 18a$^{\#}$), 0.93 (s, 3H). ^{13}C NMR (126 MHz, $CDCl_3$, $^{\#}$ denotes major diastereomer signals) δ 166.76, 165.99, 165.75 (18a*), 133.20 (18a$^{\#}$), 133.11, 133.01, 130.61, 130.35, 130.17, 129.68 (18a$^{\#}$), 129.63, 128.55 (18a$^{\#}$), 128.47, 78.35 (18a$^{\#}$), 76.15, 70.54 (18a$^{\#}$), 70.21, 63.31, 41.39, 37.46 (18a$^{\#}$), 35.39 (18a$^{\#}$), 34.69, 31.32 (18a$^{\#}$), 30.65, 30.07, 29.69, 29.59, 29.42 (18a$^{\#}$), 27.49 (18a$^{\#}$), 27.14, 26.23, 21.98 (18a$^{\#}$), 21.78, 19.37. FT-IR: ν = 3520, 2929, 2862, 1696, 1600, 1452, 1266, 1110 cm^{-1}. HR-MS: calc. for $[M+H]^+$ $C_{15}H_{21}O_3$: 249.14852 found 249.14911.

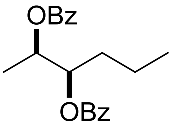

Hexane-2,3-diyl dibenzoate (20a)
major
d.r. = 3:1

+

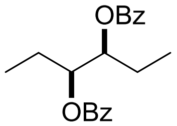

Hexane-3,4-diyl dibenzoate (20b)
minor
d.r. = 2:1

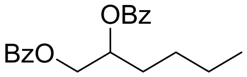

Hexane-1,2-diyl dibenzoate (20c)
minor

Regioisomeric ratio (r.r.) = 6.5:1.5:1

Dioxygenation of *n*-hexane (19)

Prepared according to the general procedure B using 2.5 equiv of NaN_3. Column chromatography (eluent: petroleum ether:EtOAc) provided the pure products. The product was obtained as colorless oil (200 mg, 0.61 mmol, 68%). R_f = 0.88 (cyclohexane : EtOAc = 1:1). r.r. = 6.5(**20a**):1.5(**20b**):1(**20c**). ^{1}H NMR (500 MHz, $CDCl_3$, $^{\#}$ denotes major-, * minor diastereomer signals) δ 8.14 – 7.91 (m, 4H), 7.62 – 7.48 (m, 2H), 7.50 – 7.35 (m, 4H), 5.51 (m, 2H, 20a*), 5.45 (dt, *J* = 9.1, 3.9 Hz, 2H, 20a$^{\#}$), 5.42 – 5.33 (m, 2H, 20b$^{\#}$), 5.26 – 5.09 (m, 2H, 20b*), 4.52 (ddd, *J* = 18.6, 11.9, 5.1 Hz, 2H, 20c), 4.52 (ddd, *J* = 18.6, 11.9, 5.1 Hz, 1H, 20c), 4.27 – 4.18 (m, 1H), 2.12 – 1.96 (m, 4H, 20c), 1.89 – 1.65 (m, 2H), 1.45 (d, *J* = 6.5 Hz, 3H, 20a*), 1.40 (d, *J* = 6.5 Hz, 3H, 20a$^{\#}$), 1.40 – 1.24 (m, 2H), 1.05 – 0.88 ppm (m, CH_3, 20a, b, c). ^{13}C NMR (126 MHz, $CDCl_3$) δ 166.32, 166.02, 133.09, 133.07, 133.06, 133.03, 130.35, 130.25, 129.78, 129.76, 129.75, 128.51, 128.48, 128.46, 128.44, 77.16, 76.91, 76.00, 75.63, 75.39, 75.12, 72.35, 71.95, 71.61, 33.00, 32.46, 27.46, 24.23, 23.31, 22.66, 18.92, 18.64, 16.75, 15.25, 14.07, 14.05, 10.08, 9.82, 9.70 ppm. FT-IR: ν = 3064, 2961, 2875, 1715, 1260, 1095 cm^{-1}.

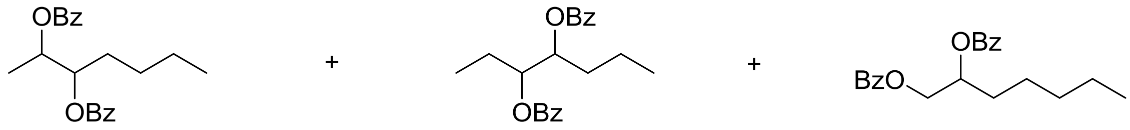

heptane-2,3-diyl dibenzoate (22a)
major
d.r. = 2:1

heptane-3,4-diyl dibenzoate (22b)
minor
d.r. = 2.5:1

heptane-1,2-diyl dibenzoate (22c)
minor

Regioisomeric ratio (r.r.) = 5.5:2:1

Dioxygenation of *n*-hepante (21)

Prepared according to the general procedure B using 2.5 equiv of NaN_3. Column chromatography (eluent: petroleum ether:EtOAc) provided products. The products were obtained as colorless oil (224 mg, 0.66 mmol, 73%). R_f = 0.87 (cyclohexane : EtOAc = 1:1). r.r. = 5.5(**22a**):2(**22b**):1(**22c**). ^{1}H NMR (500 MHz, $CDCl_3$, $^{\#}$ denotes major-, * minor diastereomer signals) δ 8.14 – 7.91 (m, 4H), 7.62 – 7.49 (m, 2H), 7.50 – 7.35 (m, 4H), 5.54 – 5.48 (m, 2H, 22b*), 5.43 (ddd, *J* = 10.5, 7.2, 2.8 Hz, 2H, 22b$^{\#}$), 5.41 – 5.36 (m, 2H, 22a$^{\#}$), 5.36 – 5.30 (m, 2H, 22a*), 4.52 (ddd, *J* = 18.6, 11.9, 5.0 Hz, 2H, 22c), 4.23 (ddd, *J* = 18.6, 11.9, 5.0 Hz, 1H, 22c), 1.90 – 1.59 (m, 2H), 1.44 (d, *J* = 6.2 Hz, 3H, 22a*), 1.40 (d, *J* = 6.2 Hz, 3H, 22a$^{\#}$), 1.39 – 1.24 (m, 4H), 1.02 – 0.96 (t, *J* = 7.2 Hz, 3H, 22b$^{\#}$), 0.94 (t, *J* = 3.7 Hz, 3H, 22b*), 0.93 – 0.86 ppm (m, 3H). ^{13}C NMR (126 MHz, $CDCl_3$) δ 166.33, 166.29, 166.16, 166.03, 133.09, 133.07, 133.06, 130.47, 130.36, 130.26, 129.80, 129.79, 129.76, 129.74, 128.52, 128.49, 128.46, 128.44, 77.16, 76.91, 76.04, 75.65, 75.36, 74.14, 71.94, 71.57, 33.27, 30.57, 30.10, 27.76, 27.45, 24.26, 22.66, 18.69, 16.76, 15.20, 14.06, 14.04, 14.02, 9.83 ppm. FT-IR: ν = 2958, 2931, 2872, 1716, 1261, 1095 cm^{-1}.

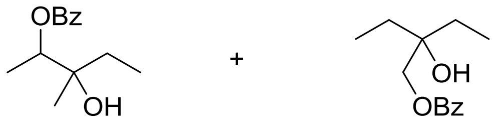

3-hydroxy-3-methylpentan-2-yl benzoate (24a)
major

2-ethyl-2-hydroxybutyl benzoate (24b)
minor

Regioisomeric ratio (r.r.) = 7.7:1

Dioxygenation of 3-methylpentane (23)

Prepared according to the general procedure B using 3.5 equiv of NaN_3. The products were obtained as colorless oil (110 mg, 0.49 mmol, 55%). R_f = 0.7 (cyclohexane : EtOAc = 1:1). r.r. = 7.7(**24a**):1(**24b**), d.r. = 1.3:1. ^{1}H NMR (500 MHz, $CDCl_3$, $^{\#}$ denotes major-, * minor diastereomer signals) *δ* 8.12 – 7.97 (m, 2H), 7.56 (t, *J* = 7.4 Hz, 1H), 7.44 (t, *J* = 7.7 Hz, 2H), 5.11 (dd, *J* = 12.5, 6.4 Hz, 1H), 4.25 (s, 2H, 22b), 1.91 (br. s, 1H), 1.71 – 1.52 (m, 2H), 1.34 (d, 6.8 Hz, 3H, 24a$^{\#}$), 1.32 (d, 6.6 Hz, 3H, 24a*), 1.23 (s, 3H, 24a$^{\#}$), 1.22 (s, 3H, 24a*), 1.01 – 0.91 ppm (m, 3H). ^{13}C NMR (126 MHz, $CDCl_3$, $^{\#}$ denotes major-, * minor diastereomer signals, $^{\sim}$ denotes minor regioisomer signals) δ ^{13}C NMR (126 MHz, $CDCl_3$, $^{\#}$ denotes major-, * minor diastereomer signals) δ 166.73 (24b), 166.21 (24a$^{\#}$), 166.08 (24a*), 133.25 (24b), 133.14, 130.46 (24b), 130.04, 129.71 (24b), 129.67, 128.54 (24b), 128.52, 76.50, 76.15, 74.43, 74.16, 74.05, 69.39, 31.71, 30.48, 28.76, 22.75, 21.69, 14.98, 14.59, 7.84, 7.77, 7.76 ppm. FT-IR: *ν* = 3496, 2974, 2941, 2882, 1699, 1269 cm^{-1}. HR-MS (**24a**): calc. for $[M+H]^+$ $C_{13}H_{19}O_3$: 223.13287 found 223.13241.

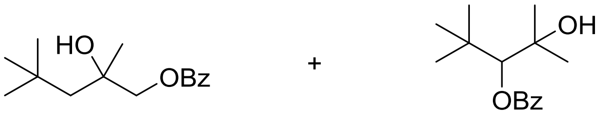

2-hydroxy-2,4,4-trimethyl-pentyl benzoate (26a) *major* | **2-hydroxy-2,4,4-trimethyl-pentan-3-yl benzoate (26b)** *minor*

Regioisomeric ratio (r.r.) = 8:1

Dioxygenation of 2,2,4-Trimethylpentane (25)

Prepared according to the general procedure B using 3.5 equiv of NaN_3. The products were obtained as colorless oil (45 mg, 0.18 mmol, 20%). R_f = 0.72 (cyclohexane : EtOAc = 1:1). r.r. = 8(**26a**):1(**26b**). ^{1}H NMR (500 MHz, $CDCl_3$) δ 8.13 – 7.97 (m, 2H), 7.58 (d, *J* = 7.4 Hz, 1H), 7.47 (t, *J* = 7.7 Hz, 2H), 4.23 (dd, *J* = 38.5, 11.0 Hz, 2H), 3.49 (s, 1H, 26b), 1.63 (q, *J* = 14.8 Hz, 2H), 1.39 (s, 2H), 1.27 (m, 2H), 1.10 (s, 9H). ^{13}C NMR (126 MHz, $CDCl_3$) δ 166.66, 133.32, 130.08, 129.74, 128.62, 73.43, 72.78, 51.18, 31.68, 31.36, 26.16. FT-IR: ν = 3489, 2953, 2360, 2105, 1718, 1270, 1111 cm^{-1}. HR-MS (**24a**): calc. for $[M+H]^+$ $C_{15}H_{23}O_3$: 251.16417 found 251.16373.

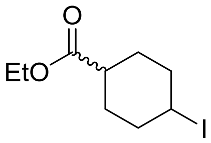

Synthesis of ethyl 4-iodocyclohexane-1-carboxylate (27)

Prepared according to reference 5. To a stirring solution of ethyl 4-hydroxycyclohexane-1-carboxylate (344 mg, 2 mmol, 1 equiv) in dichloromethane (8 mL) triphenylphosphine (788 mg, 1.5 mmol, 1.5 equiv), imidazole (204 mg, 1.5 mmol, 1.5 equiv) and iodine (762 mg, 1.5 mmol, 1.5 equiv) were successively added and the reaction was stirred for 3 h at rt. Afterwards, the reaction was diluted with DCM (20 mL) and quenched with saturated Na_2SO_3 solution (10 mL). The aqueous phase was extracted two times with dichloromethane (2x20 mL), the combined organic layers were dried over $MgSO_4$ and concentrated under reduced pressure. Column chromatography provided the pure product (eluent: petroleum ether). The product was obtained as colorless oil (276 mg, 0.98 mmol, 49%). R_f = 0.55 (cyclohexane : EtOAc = 10:1). d.r. = 2.5:1. ^{1}H NMR (500 MHz, $CD_2Cl_2$$^{\#}$ denotes major-, * minor diastereomer signals) δ 4.68$^{\#}$ (s, 1H), 4.17* (td, *J* = 7.6, 3.8 Hz, 1H), 4.09 (m, 2H), 2.43 – 2.31 (m, 1H), 2.14 – 1.70 (m, 6H), 1.53 (2H), 1.28 – 1.18 (m, 3H). ^{13}C NMR (126 MHz, CD_2Cl_2) δ 175.15, 60.83, 42.01, 39.53, 36.44, 33.98, 31.26, 26.73, 14.57, 14.52.

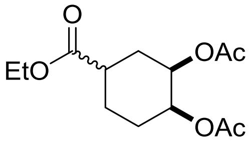

Synthesis of 4-(ethoxycarbonyl)cyclohexane-1,2-diyl diacetate (28)

To a stirring solution of 4-MeC_6H_4I (66.7 mg, 0.3 mmol, 0.6 equiv) and AcO_2H (704 µL of a 39% solution in AcOH, 3.75 mmol, 7.35 equiv) in glacial acetic acid (1.8 mL) ethyl 4-iodocyclohexane-1-carboxylate (254 mg, 0.5 mmol, 1 equiv) was added portionwise over a time period of 15 minutes under waterbath cooling. After that time, the reaction was stirred for 24 h at rt. The reaction was diluted with DCM (20 mL) and slowly neutralized with 1 M NaOH (10 mL). The aqueous phase was extracted two times with dichloromethane (2x20 mL), the combined organic layers were dried over $MgSO_4$ and concentrated under reduced pressure. The crude reaction was dissolved in DCM-Py (1:1, 3 mL) and 4-(dimethylamino)-pyridine (2.75 mg, 5 mol%, 0.025 mmol) was added. Ac_2O (144 µL, 1.5 mmol, 3 equiv) was added dropwise at 0 °C and the reaction was stirred at rt for 24 h. Column chromatography (eluent: petroleum ether : EtOAc) provided the pure products. The reaction was diluted with DCM (20 mL) and washed with 1 M HCl solution. The aqueous phase was extracted two times with dichloromethane (2x20 mL), the combined organic layers were dried over $MgSO_4$ and concentrated under reduced pressure. Column chromatography (eluent: petroleum ether : EtOAc) provided the pure product. The product was obtained as colorless oil (119 mg, 0.44 mmol, 86%). R_f = 0.78 (cyclohexane : EtOAc = 1:1). *cis* (major) : *trans* (minor) = 2.5:1. ^{1}H NMR (600 MHz, $CDCl_3$, $^{\#}$ denotes major-, * minor diastereomer signals) δ 5.34 – 5.27* (m, 1H), 5.24 – 5.17* (m, 1H), 5.00$^{\#}$ (dd, *J* = 9.1, 5.6 Hz, 1H), 4.88 – 4.79$^{\#}$ (m, 3H), 4.13 (m, 2H), 2.69 – 2.22 (m, 1H), 2.20 – 1.94 (m, 7H), 1.75 (m, 4H), 1.30 – 1.18 (m, 3H). ^{13}C NMR (151 MHz, $CDCl_3$) δ 174.98, 174.90, 174.34, 173.92, 170.73, 170.68, 170.64, 170.58, 170.46, 170.32, 170.04, 73.44, 72.98, 71.55, 71.44, 69.96, 69.50, 68.85, 68.81, 61.05, 60.96, 60.95, 60.83, 43.73, 43.02, 41.11, 40.93, 37.98, 37.54, 33.14, 32.69, 31.42, 30.17, 30.10, 29.41, 29.33, 28.67, 27.89, 26.71, 26.41, 25.86, 25.51, 23.72, 22.74, 21.46, 21.45, 21.43, 21.40, 21.37, 21.33, 14.54, 14.52, 14.49. FT-IR: ν = 2955, 1729, 1444, 1367, 1227, 1107, 1028 cm^{-1}. HR-MS: calc. for $[M+H]^+$ $C_{13}H_{21}O_6$: 273.13326 found 273.13333.

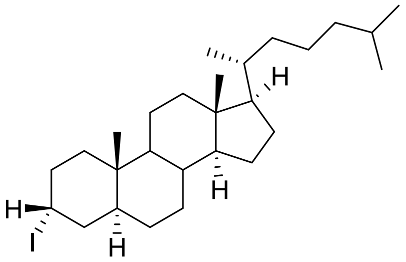

Synthesis of 3α-iodo-5α-cholestane (29)

Prepared according to reference 5. To a stirring solution of 5α-cholestane-3β-ol (389 mg, 1 mmol, 1 equiv) in dichloromethane (4 mL) triphenylphosphine (394 mg, 1.5 mmol, 1.5 equiv), imidazole (102 mg, 1.5 mmol, 1.5 equiv) and iodine (381 mg, 1.5 mmol, 1.5 equiv) were successively added and the reaction was stirred for 3 h at rt. Afterwards, the reaction was diluted with DCM (20 mL) and quenched with saturated Na_2SO_3 solution (10 mL). The aqueous phase was extracted two times with dichloromethane (2x20 mL), the combined organic layers were dried over $MgSO_4$ and concentrated under reduced pressure. Column chromatography provided the pure product (eluent: petroleum ether). The product was obtained as pale yellow solid (339 mg, 0.68 mmol, 68%). R_f = 0.9 (cyclohexane : EtOAc = 10:1). d.r. > 20:1. ^{1}H NMR (500 MHz, $CDCl_3$) δ 4.95 (s, 1H), 2.02 – 1.75 (m, 3H), 1.66 (m, 4H), 1.49 (m, 6H), 1.28 (m, 8H), 1.18 – 0.92 (m, 9H), 0.90 (d, *J* = 6.6 Hz, 3H), 0.86 (dd, *J* = 6.6, 2.5 Hz, 6H), 0.82 (m, 1H), 0.79 (s, 3H), 0.64 ppm (s, 3H). ^{13}C NMR (126 MHz, $CDCl_3$) δ 56.54, 56.35, 53.88, 42.69, 42.13, 40.05, 39.63, 38.88, 38.59, 36.66, 36.28, 35.97, 35.53, 34.47, 32.85, 31.90, 28.39, 28.15, 27.89, 24.30, 24.00, 22.99, 22.72, 20.91, 18.79, 13.53, 12.21 ppm.

Dioxygenation of 3α-iodo-5α-cholestane (29)

To a stirring solution of 4-MeC_6H_4I (66.7 mg, 0.3 mmol, 0.6 equiv) and AcO_2H (704 µL of a 39% solution in AcOH, 3.75 mmol, 7.35 equiv) in glacial acetic acid (1.8 mL) 3α-iodo-5α-cholestane (254 mg, 0.5 mmol, 1 equiv) was added portionwise over a time period of 15 minutes under waterbath cooling. After that time, the reaction was stirred for 24 h at rt. The reaction was diluted with DCM (20 mL) and slowly neutralized with 1 M NaOH (10 mL). The aqueous phase was extracted two times with dichloromethane (2x20 mL), the combined organic layers were dried over $MgSO_4$ and concentrated under reduced pressure. The crude mixture was dissolved in 5 mL MeOH, whereupon LiOH (24 mg, 1 mmol, 2 equiv) was added and the reaction was stirred at room temperature. After completion, HCl (1.25 M) in MeOH (0.85 mL) was added and MeOH was removed under reduced pressure. Column chromatography (eluent: petroleum ether : EtOAc) provided the pure products in the following order:

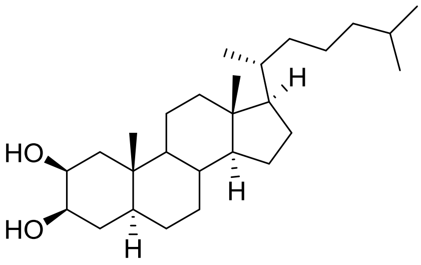

cholestane-2β,3β-diol (30)
cis : *trans* = 5:1

The product was obtained as white solid (52 mg, 0.13 mmol, 25%). R_f = 0.38 (cyclohexane : EtOAc = 1:1). *cis* (2β,3β) : *trans* (2α,3β) = 5:1. GC-MS: calculated exact mass: 404.36 found 404.4. ^{1}H NMR (500 MHz, $CDCl_3$, $^{\#}$ denotes major-, * minor diastereomer signals) δ 4.01$^{\#}$ (d, *J* = 2.8 Hz, 1H), 3.73* (m, 1H), 3.62$^{\#}$ (dt, *J* = 11.3, 4.1 Hz, 1H), 3.58 – 3.50* (m, 1H), 2.35 (br. s, 2H), 2.00 (dd, *J* = 43.9, 15.0 Hz, 2H), 1.85 – 1.42 (m, 7H), 1.43 – 0.91 (m, 27H), 0.92 – 0.77 (m, 12H), 0.64 (s, 3H), 0.61 – 0.51 ppm (m, 1H). ^{13}C NMR (126 MHz, $CDCl_3$) δ 74.92, 72.54, 72.45, 70.32, 56.66, 56.51, 56.36, 56.33, 55.32, 48.90, 45.44, 43.26, 42.73, 42.69, 40.14, 40.00, 39.62, 36.98, 36.27, 35.91, 35.58, 35.50, 35.35, 34.93, 32.56, 32.51, 32.08, 29.85, 28.46, 28.37, 28.14, 26.01, 25.98, 24.34, 24.30, 23.95, 22.97, 22.70, 21.41, 20.70, 18.77, 14.79, 14.69, 14.29, 12.22, 12.19 ppm. The spectral data are matching with reported.[6] FT-IR: *ν* = 3387, 2926, 2865, 2359, 1711, 1457, 1382, 1078, 1051 cm^{-1}.

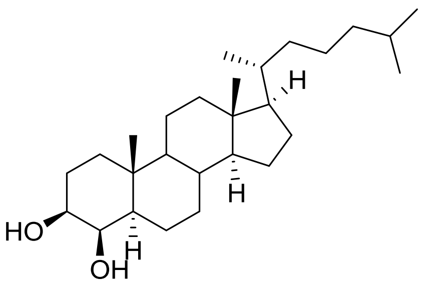

cholestane-3β,5β-diol (31)
cis : *trans* = 8.5:1

The product was obtained as white solid (28 mg, 0.07 mmol, 15%). R_f = 0.24 (cyclohexane : EtOAc = 1:1). *cis* (3β,4β): *trans* (3α,4β) = 8.5:1. GC-MS: calculated exact mass: 404.36 found 404.4. ^{1}H NMR (500 MHz, $CDCl_3$, $^{\#}$ denotes major-, * minor diastereomer signals) δ 3.42$^{\#}$ (d, *J* = 1.9 Hz, 1H), 3.37$^{\#}$ (d, *J* = 2.4 Hz, 1H), 3.26* (m, 1H), 3.12* (m, 1H), 1.58 (m, 1H), 1.35 (m, 4H), 1.23 – 0.79 (m, 15H), 0.79 – 0.39 (m, 22H), 0.27 ppm (s, 4H). ^{13}C NMR (126 MHz, $CDCl_3$, $^{\#}$ denotes major-, * minor diastereomer signals) δ 74.89*, 70.47$^{\#}$, 69.46*, 69.35$^{\#}$, 56.16, 56.04, 55.77, 54.81, 54.72, 43.32, 42.07, 42.00, 39.61, 39.48, 39.23, 38.97, 38.32, 35.63, 35.33, 35.05, 34.45, 31.96, 31.53, 31.36, 30.71, 27.75, 27.67, 27.44, 24.84, 23.55, 23.24, 21.91, 21.65, 20.29, 19.67, 17.84, 13.37, 13.25, 11.27, 11.22 ppm. Assigned according to reported spectra.[6,7] FT-IR: ν = 3378, 2926, 2865, 2359, 1711, 1456, 1382, 1078, 1050 cm^{-1}.

Control experiments

All control experiments were performed according to the optimized conditions described in the manuscript (table 2).

A) To prove the requirement of used reagents

a. without ArI

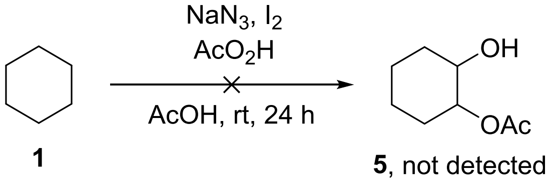

b. without NaN_3

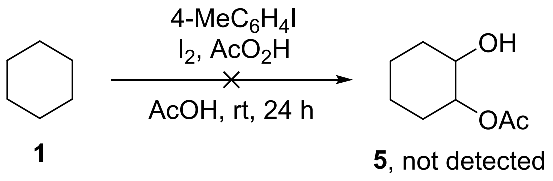

c. without I_2

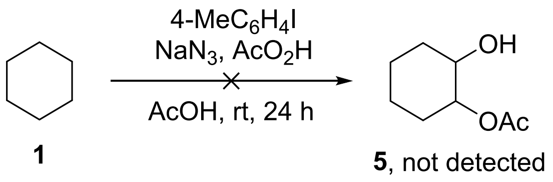

B) To prove iodocyclohexane as intermediate in the course of reaction

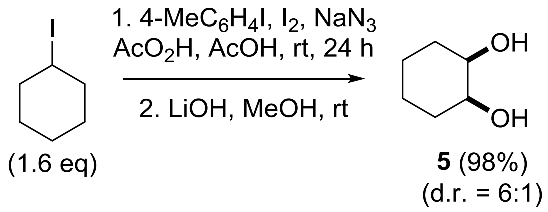

C) To prove the outcome of the relative stereochemistry to be independent of epoxidation and ring opening:

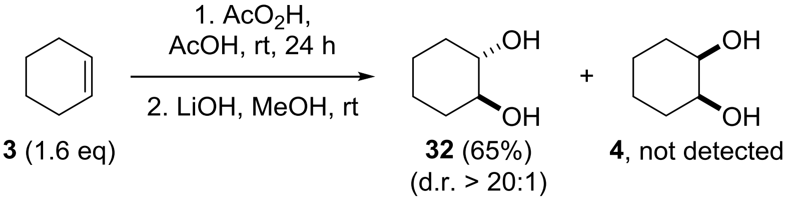

Reaction profile of the 1,2-dihydroxylation of cyclohexane

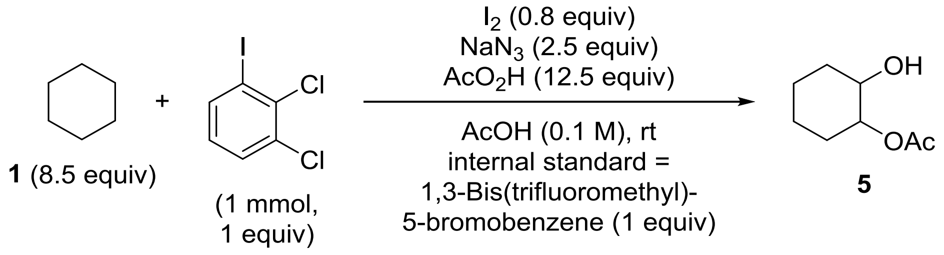

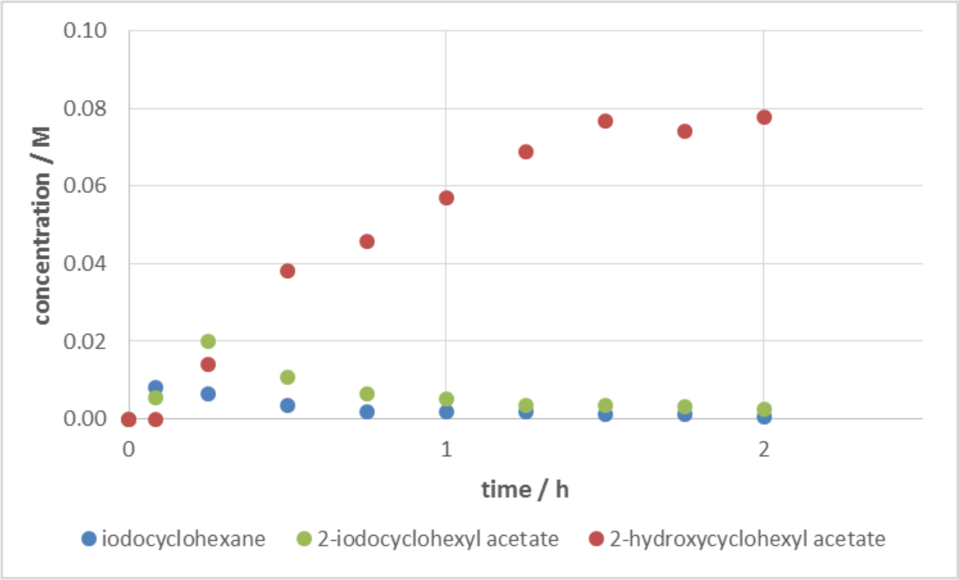

Fig. 1: Reaction profile of the 1,2-dioxygenation of cyclohexane over the course of 2.5 h.

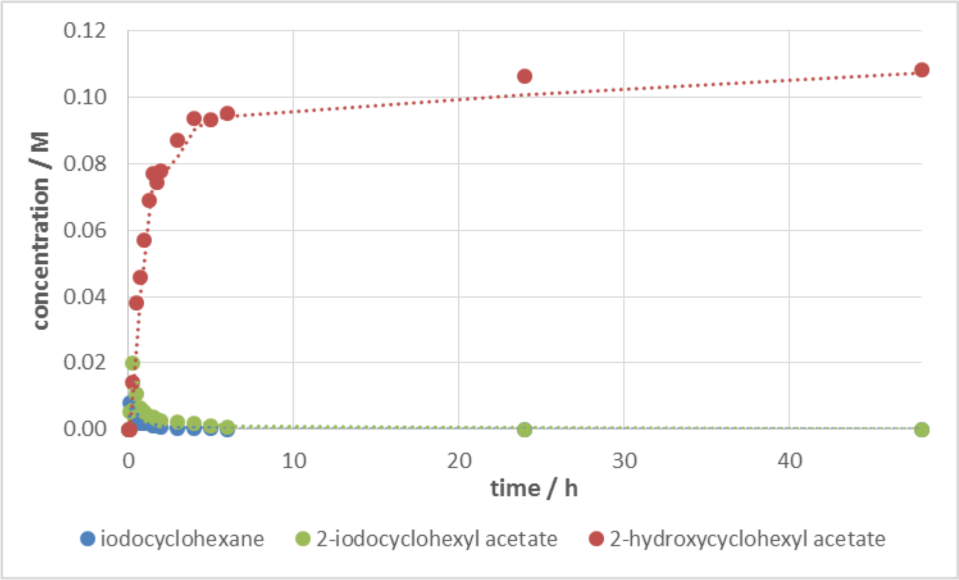

Fig. 2: Reaction profile of the 1,2-dioxygenation of cyclohexane over the course of 48 h.

Kinetic isotope effect

KIE Scheme:

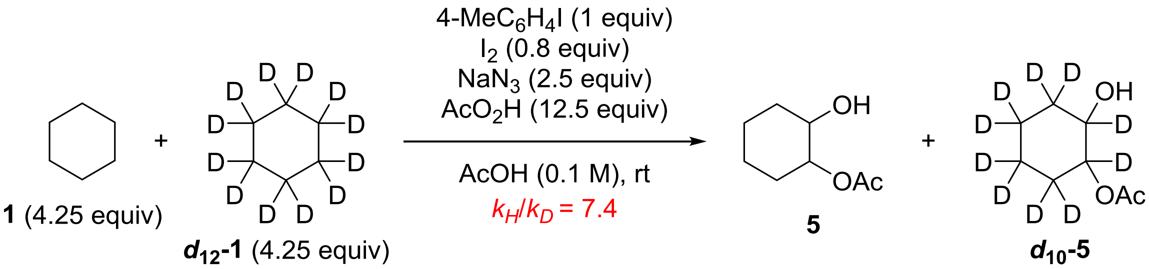

a) Full Spectrum

b) Zoom

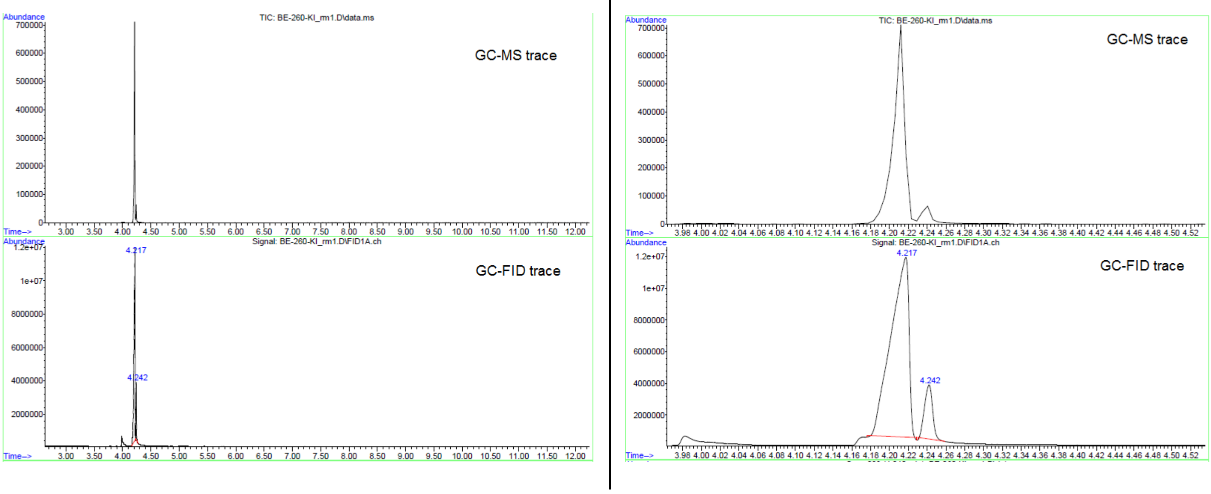

Fig. 3: GC-MS-FID spectrometry for the determination of the kinetic isotope effect for the synthesis of 2-hydroxycyclohexyl acetate (**5**) using cyclohexane (**1**) and d_{12}-cyclohexane (***d***$\boldsymbol{d_{12}}$**-1**). a) Full spectrum of the GC-MS-FID measurement. b) Zoom of the product distribution of 2-hydroxycyclohexyl acetate (**5**; t = 4.217) and d_{10}-2-hydroxycyclohexyl acetate ($\boldsymbol{d_{10}}$**-5**; t = 4.242).

Note: The kinetic isotope effect was determined using the GC-FID trace. The peak with retention time 4.217 was identified as 2-hydroxycyclohexyl acetate (**5**) and the peak with retention time 4.242 was identified as d_{10}-2-hydroxycyclohexyl acetate ($\boldsymbol{d_{10}}$**-5**). The ratio of the peak integrals (*AUC*) in the FID trace allowed the calculation of the kinetic isotope effect according to the following equation:

$$k_H/k_D = AUC_5\ (t = 4.217)/AUC_{\boldsymbol{d10\text{-}5}}\ (t = 4.242) = (1.58*10^8)/(2.14*10^7) = 7.4$$

KIE Scheme:

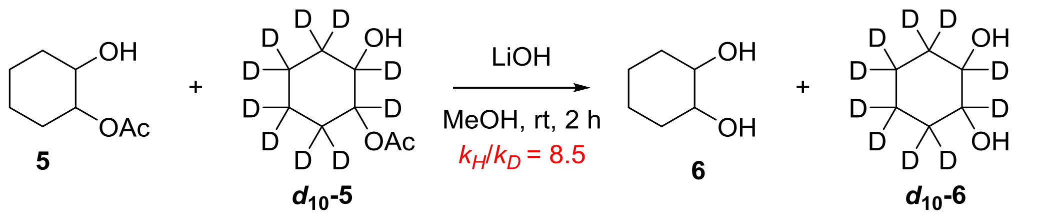

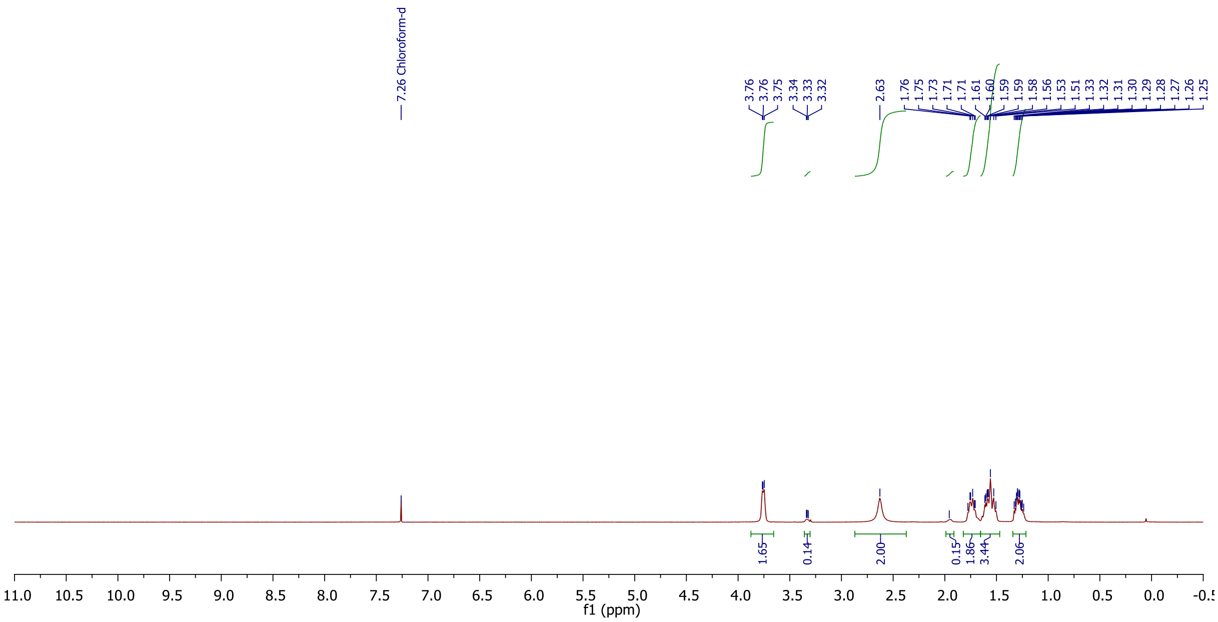

Note: The value of k_H/k_d was calculated from the ^{1}H NMR spectra above which should be the mixture of compound **6** and ***d*$_{10}$-6** (the KIE scheme). The sum of the integral of **6** and ***d*$_{10}$-6** at chemical shift 2.63 was integrated as 2.00 (both **6** and ***d*$_{10}$-6** keep the same hydroxyl groups). In total, compound **6** contained 2 hydrogen atoms at chemical shift 3.76 – 3.75 (*cis*) and 3.34 – 3.32 (*trans*), while ***d*$_{10}$-6** has no H atoms. The amount of **6** could be defined as 0.895 (1.79/2 = 0.895), on the other hand, the sum of **6** and ***d*$_{10}$-6** is 1.00, so the amount of **6** is 0.105 (1.00-0.895 = 0.105). As a result, k_H/k_D =0.895/0.105 = 8.5.

Copies of ^{1}H and ^{13}C NMR spectra

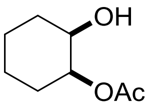

5

^{1}H NMR spectrum of isomeric mixture of 2-hydroxycyclohexyl acetate (**5**).

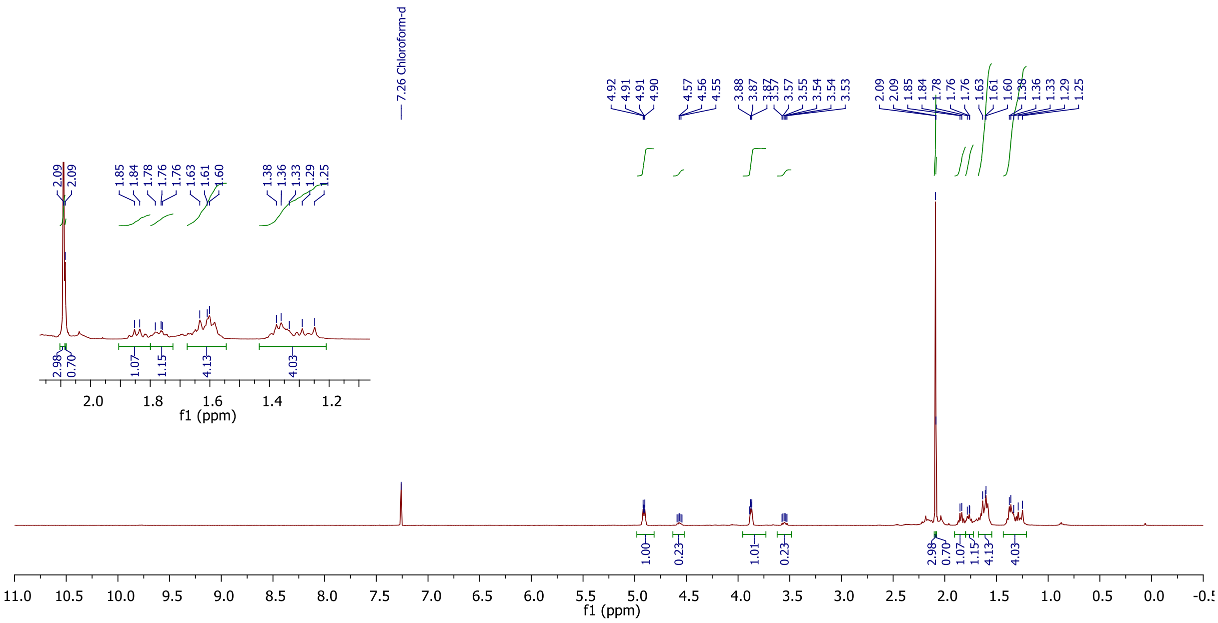

^{13}C NMR spectrum of isomeric mixture of 2-hydroxycyclohexyl acetate (**5**).

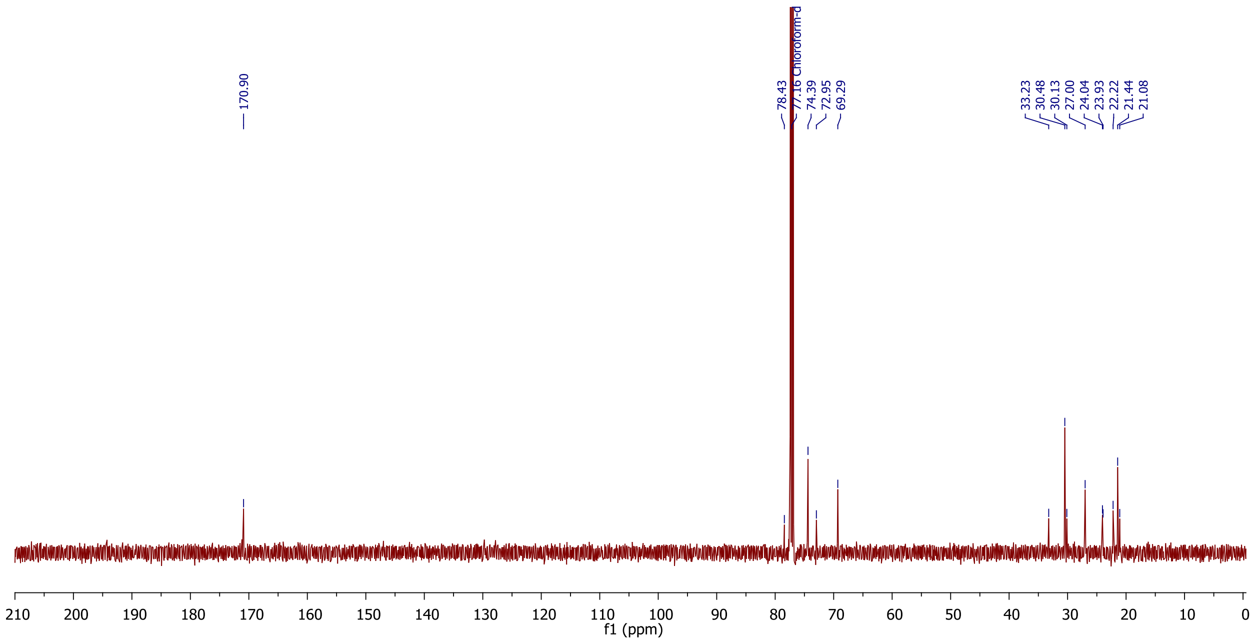

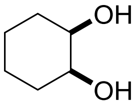

6

^{1}H NMR spectrum of *cis*-cyclohexane-1,2-diol (**6**).

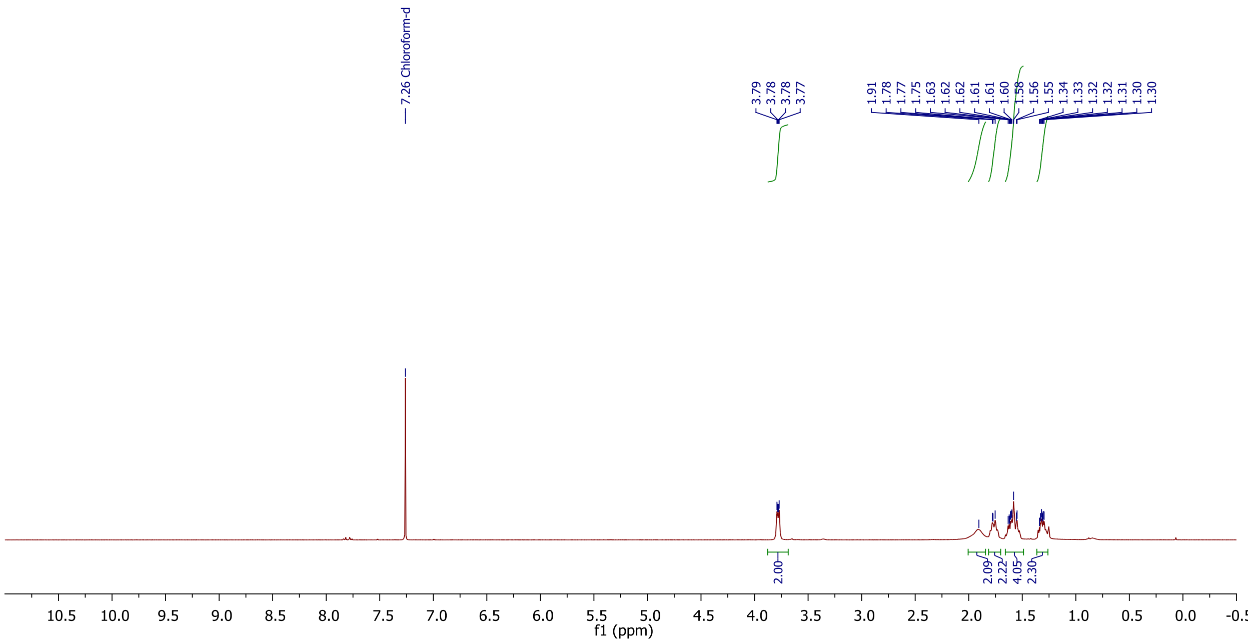

^{13}C NMR spectrum of *cis*-cyclohexane-1,2-diol (**6**).

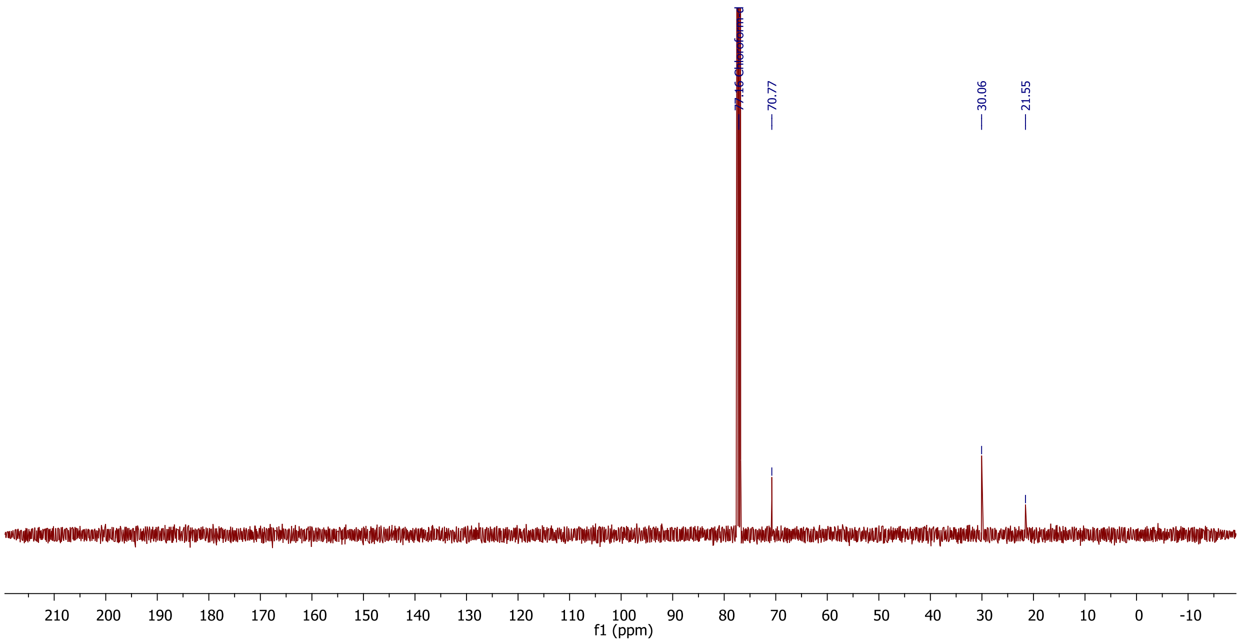

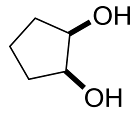

8

^{1}H NMR spectrum of *cis*-cyclopentane-1,2-diol (**8**).

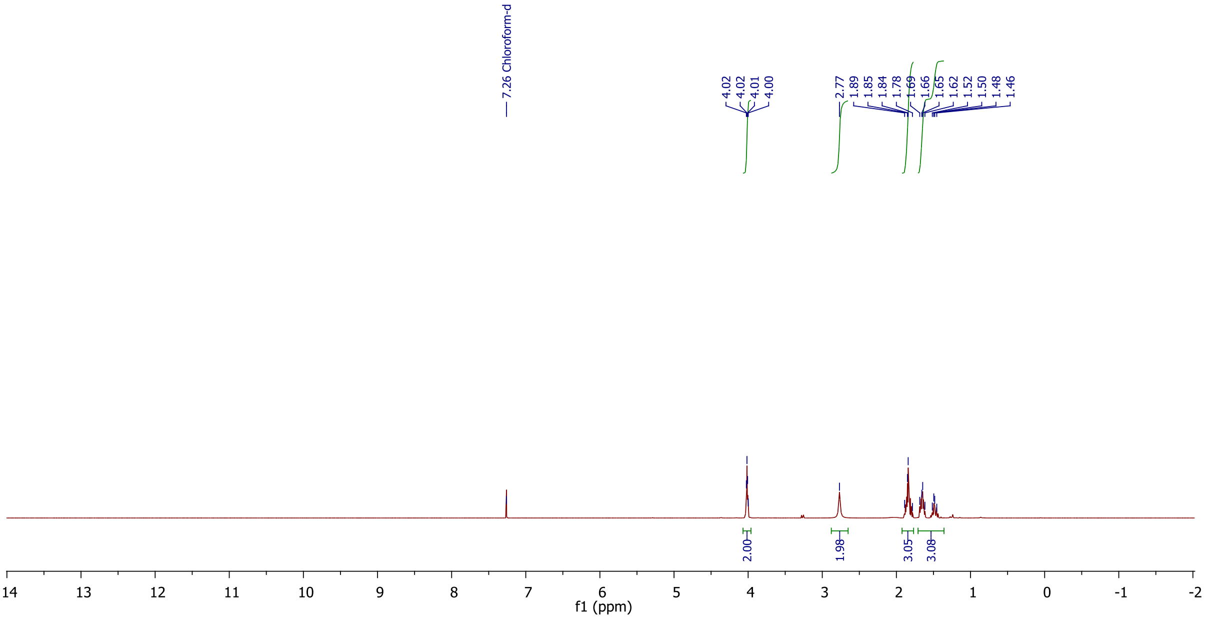

^{13}C NMR spectrum of *cis*-cyclopentane-1,2-diol (**8**).

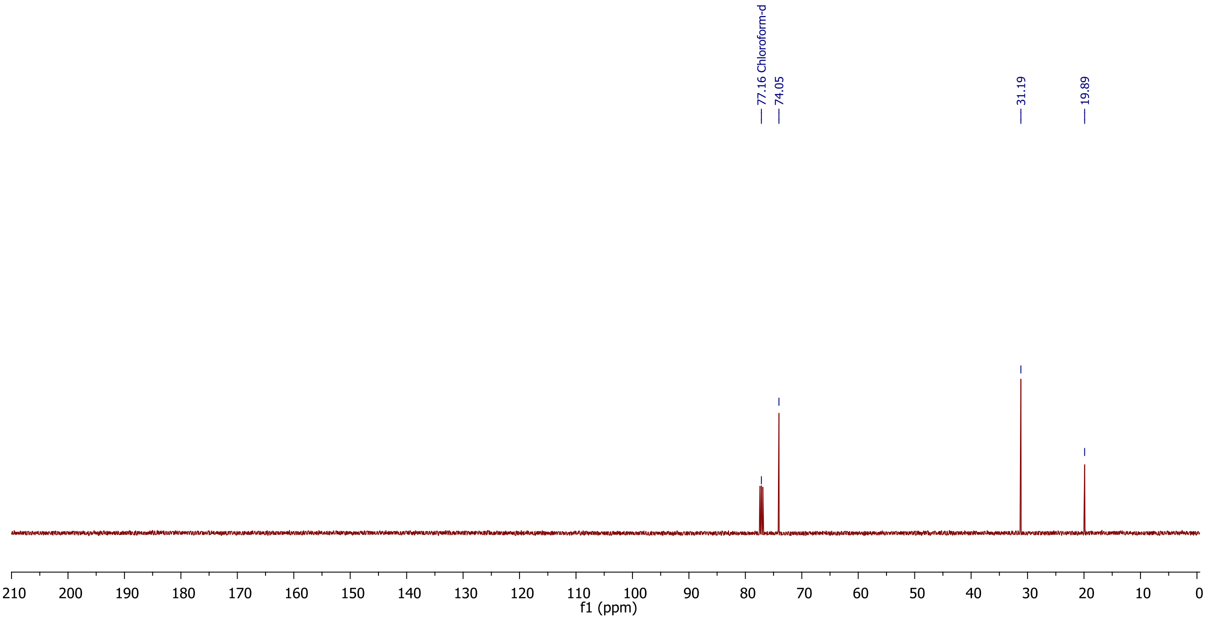

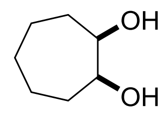

10

^{1}H NMR spectrum of *cis*-cycloheptane-1,2- diol (**10**).

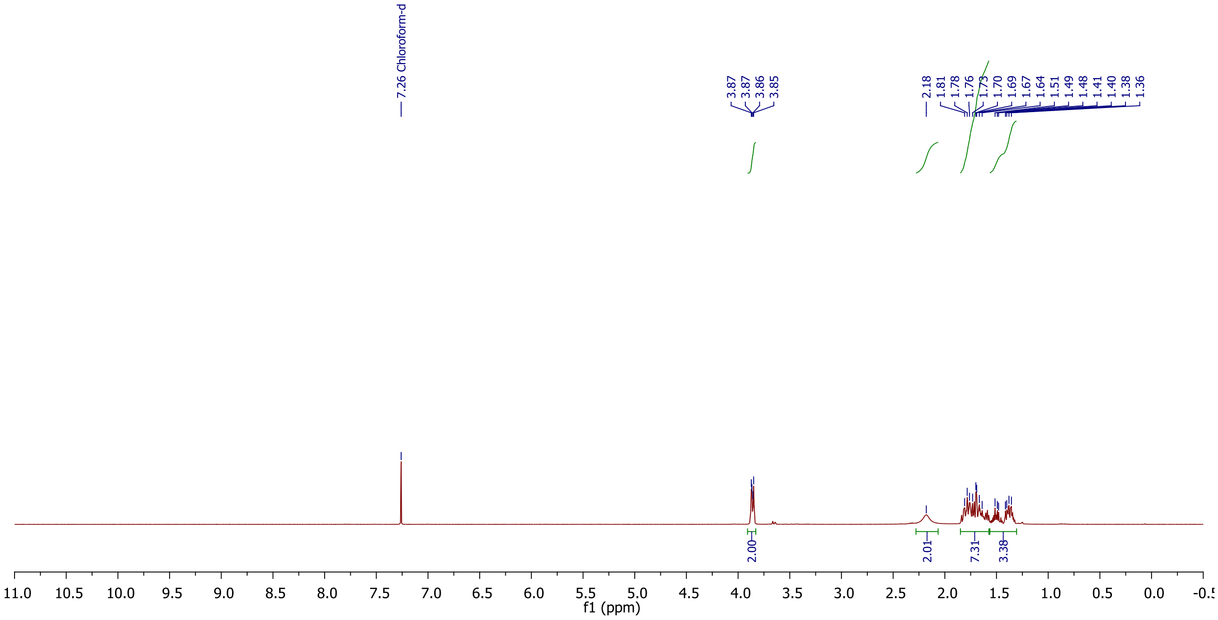

^{13}C NMR spectrum of *cis*-cycloheptane-1,2- diol (**10**).

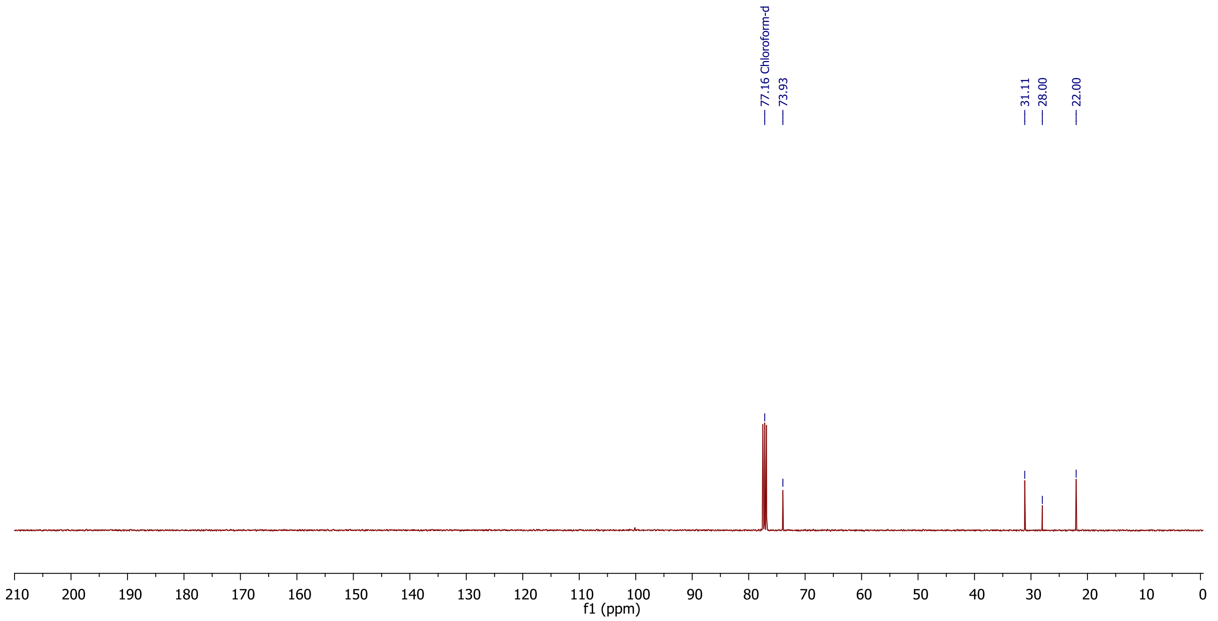

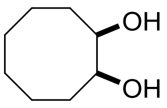

12

^{1}H NMR spectrum of *cis*-cyclooctane-1,2-diol (**12**).

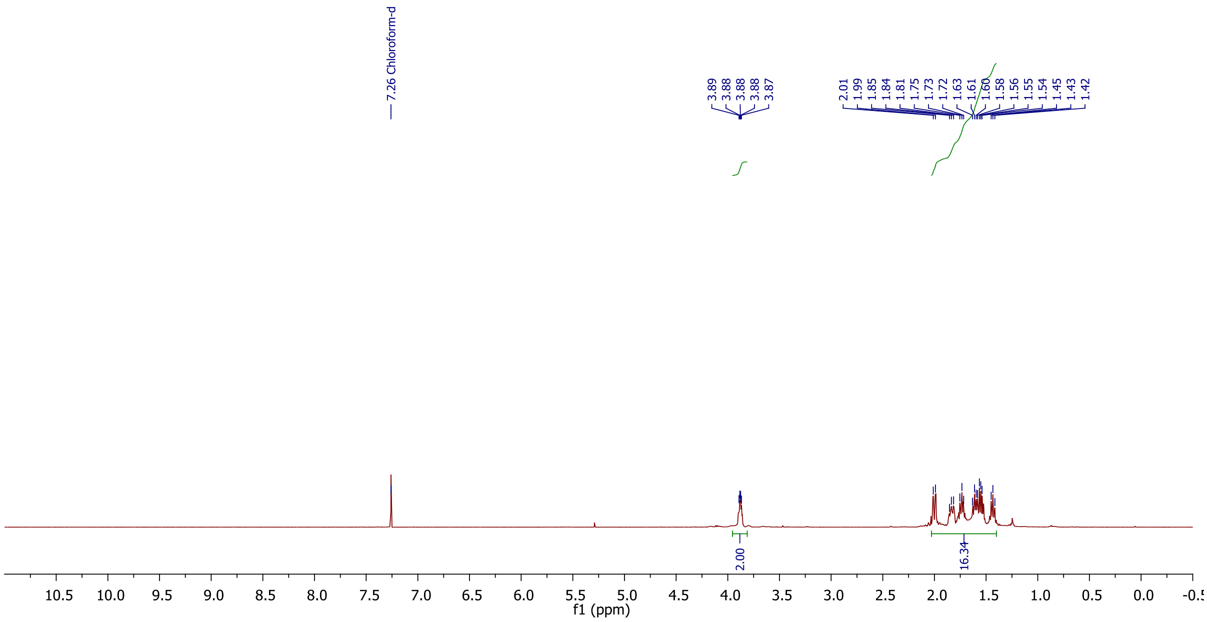

^{13}C NMR spectrum of *cis*-cyclooctane-1,2-diol (**12**).

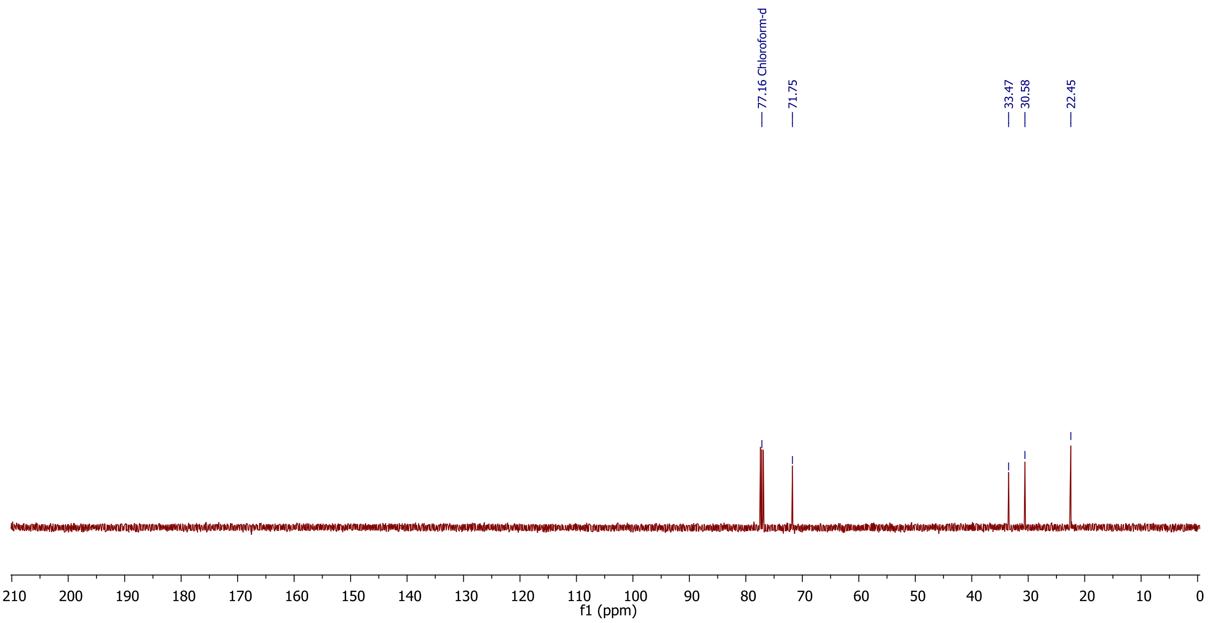

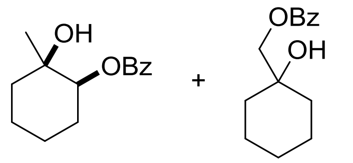

14a **14b**

Regioisomeric ratio (r.r.) > 20:1

^{1}H NMR spectrum of isomeric mixture of (1-hydroxycyclohexyl)methyl benzoate (**14a**) and (1S,2R)-2-hydroxy-2-methylcyclohexyl benzoate (**14b**).

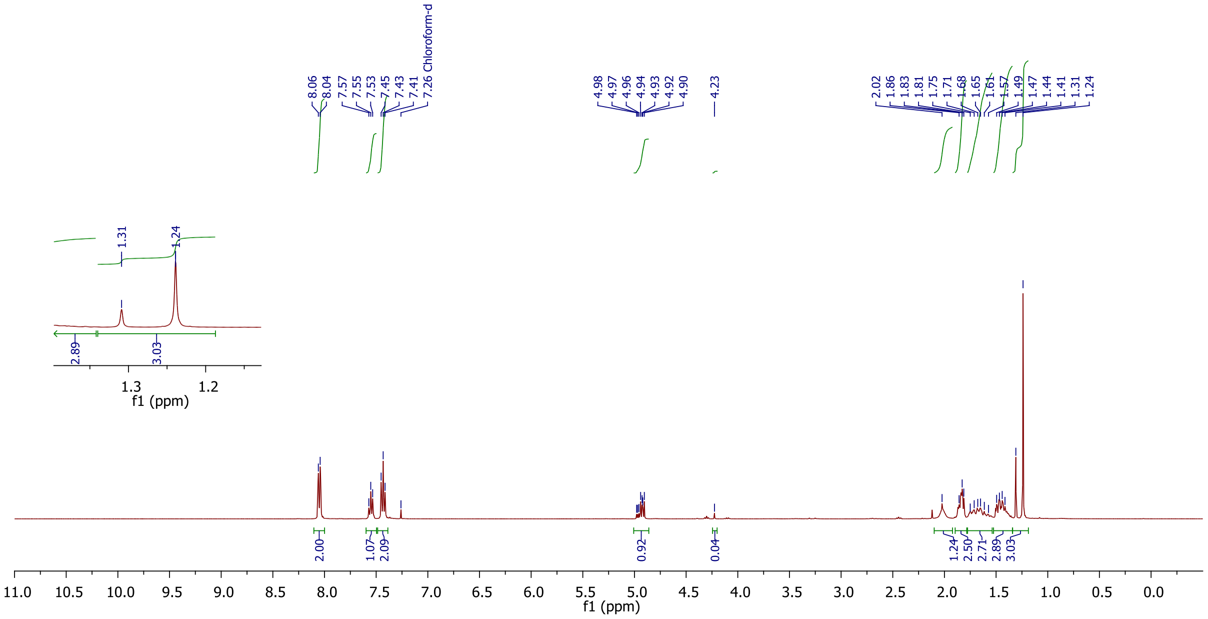

^{13}C NMR spectrum of isomeric mixture of (1-hydroxycyclohexyl)methyl benzoate (**14a**) and (1S,2R)-2-hydroxy-2-methylcyclohexyl benzoate (**14b**).

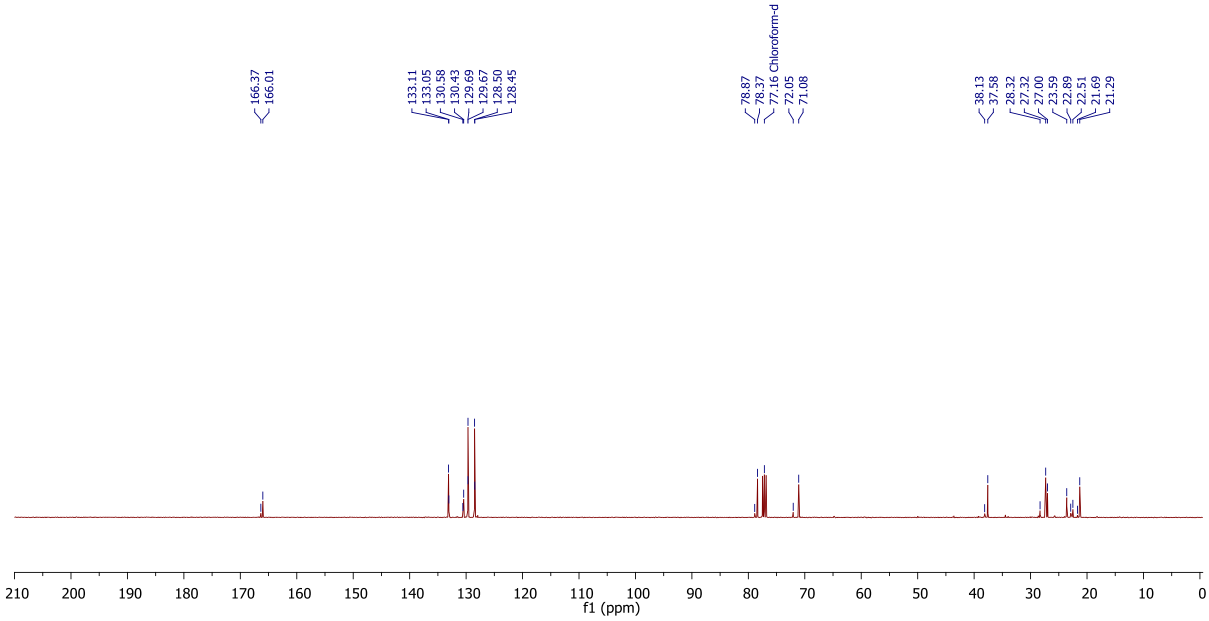

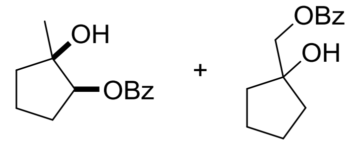

16a **16b**

Regioisomeric ratio (r.r.) = 18.5:1

^{1}H NMR spectrum of isomeric mixture of (1-hydroxycyclopentyl)methyl benzoate (**16a**) and 2-hydroxy-2-methylcyclopentyl benzoate (**16b**).

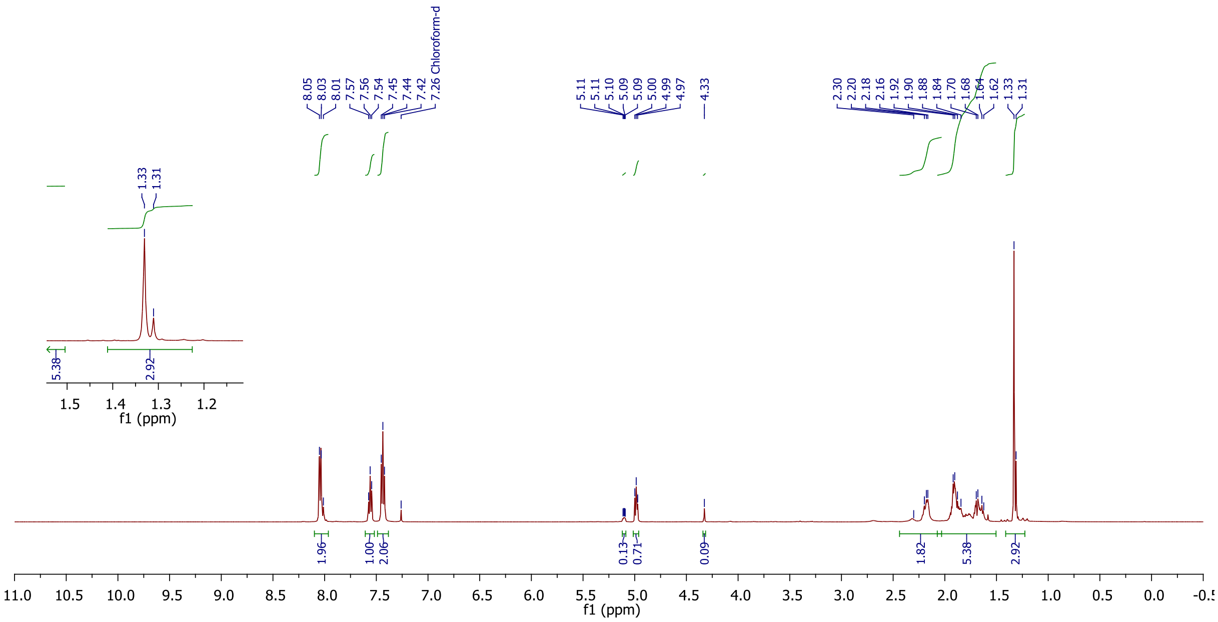

^{13}C NMR spectrum of isomeric mixture of (1-hydroxycyclopentyl)methyl benzoate (**16a**) and 2-hydroxy-2-methylcyclopentyl benzoate (**16b**).

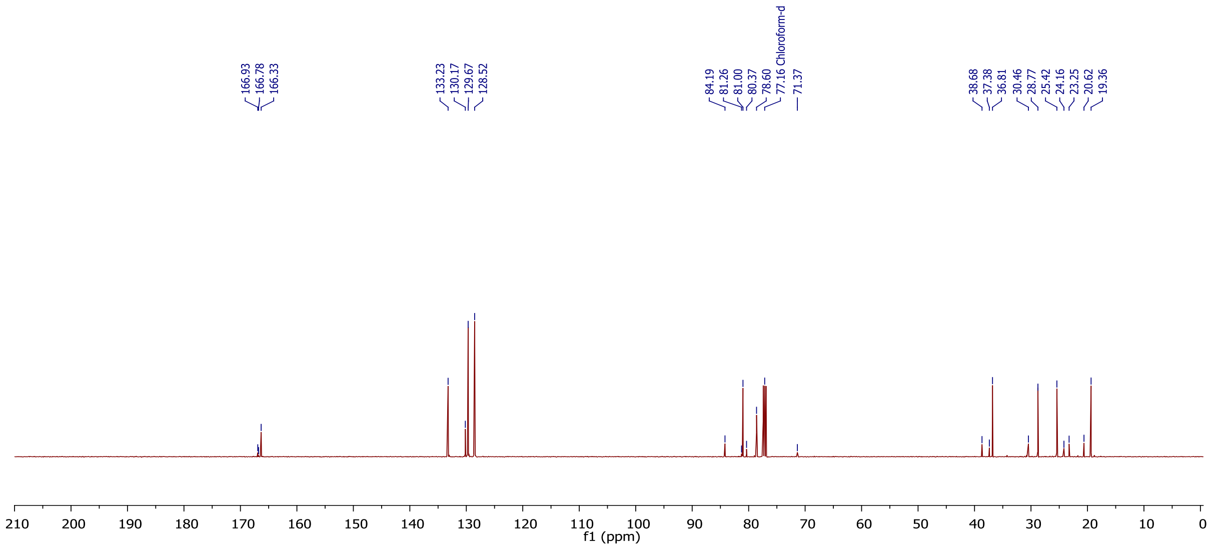

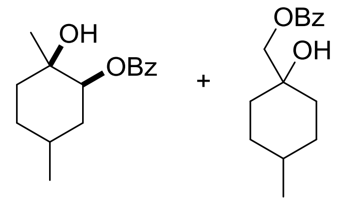

18a **18b**

Regioisomeric ratio (r.r.) > 20:1

^{1}H NMR spectrum of isomeric mixture of 2-hydroxy-2,5-dimethylcyclohexyl benzoate (**18a**) with (1-hydroxy-4-methylcyclohexyl)methyl benzoate compound (**18b**).

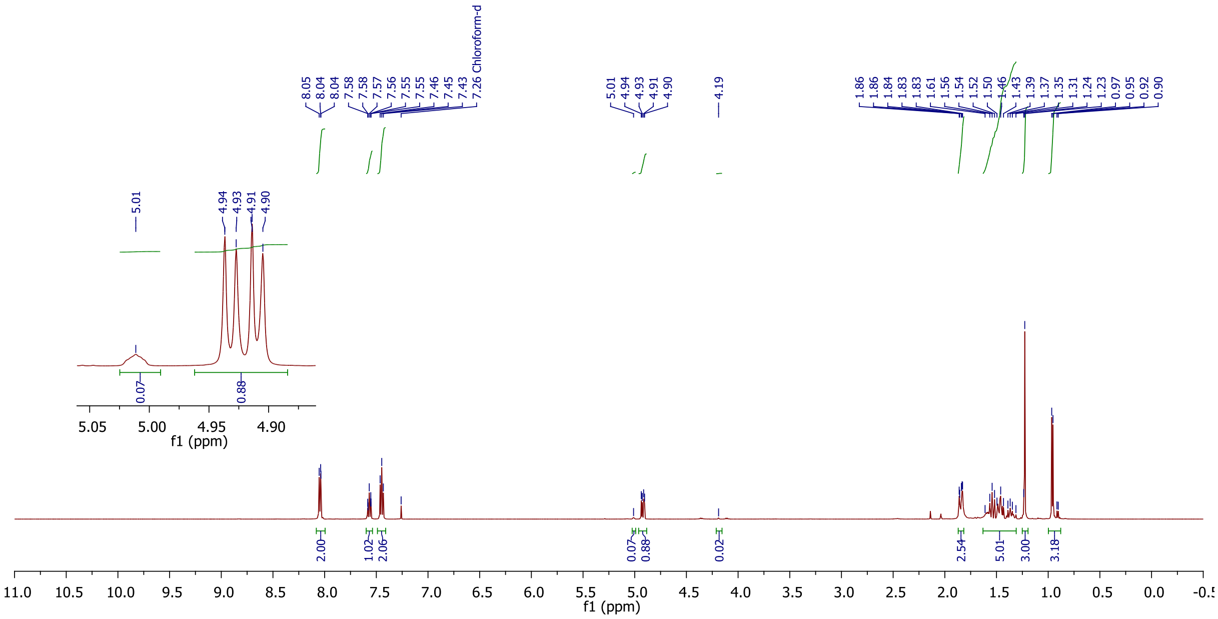

^{13}C NMR spectrum of isomeric mixture of 2-hydroxy-2,5-dimethylcyclohexyl benzoate (**18a**) with (1-hydroxy-4-methylcyclohexyl)methyl benzoate compound (**18b**).

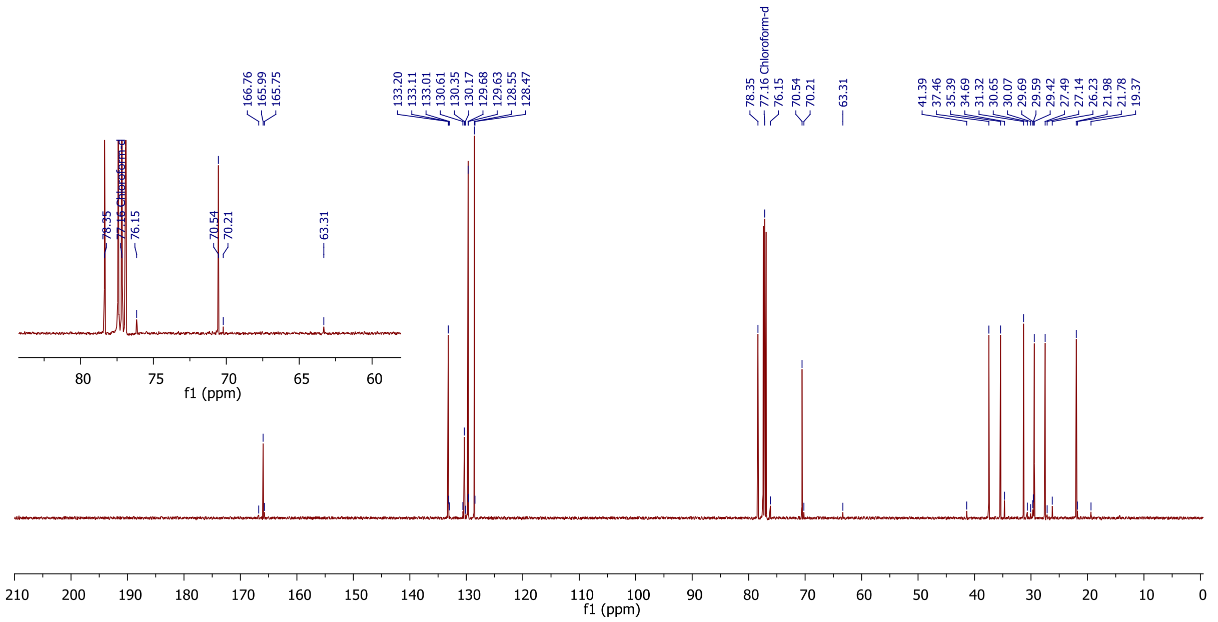

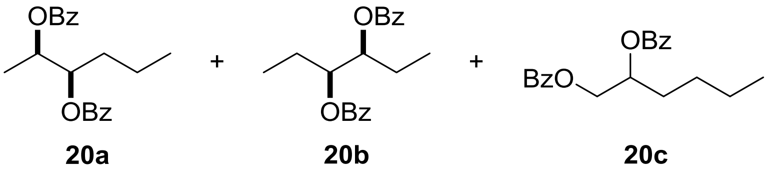

Regioisomeric ratio (r.r.) = 6.5:1.5:1

^{1}H NMR spectrum of isomeric mixture of hexane-2,3-diyl dibenzoate (**20a**), hexane-3,4-diyl dibenzoate (**20b**) and hexane-1,2-diyl dibenzoate (**20c**).

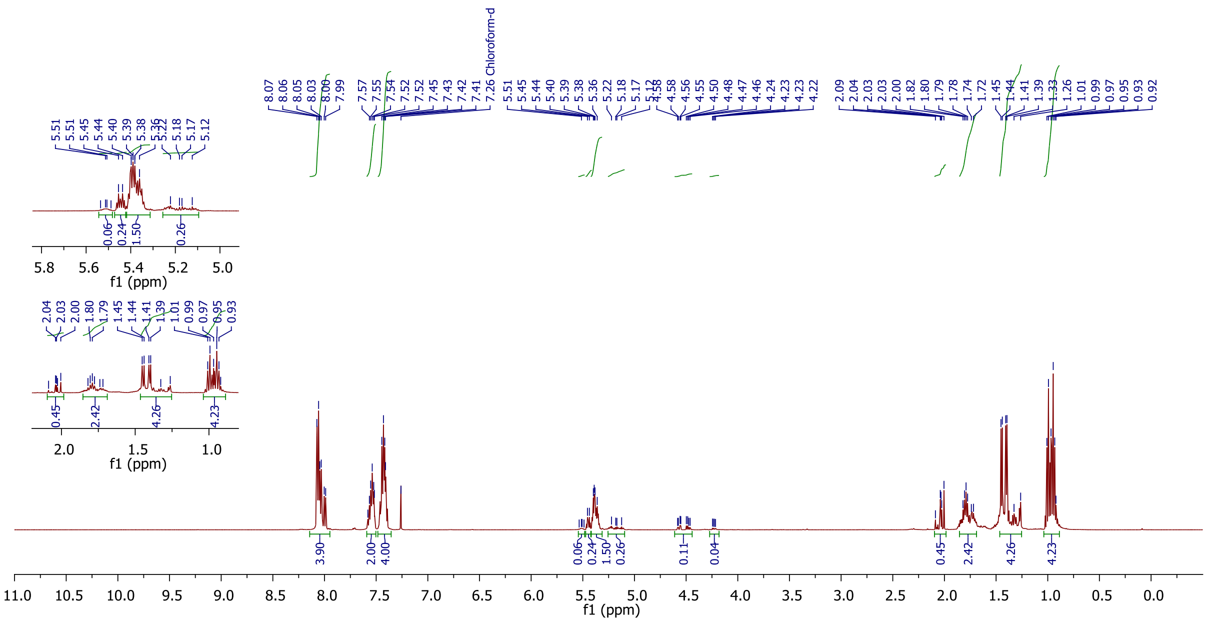

^{13}C NMR spectrum of isomeric mixture of hexane-2,3-diyl dibenzoate (**20a**), hexane-3,4-diyl dibenzoate (**20b**) and hexane-1,2-diyl dibenzoate (**20c**).

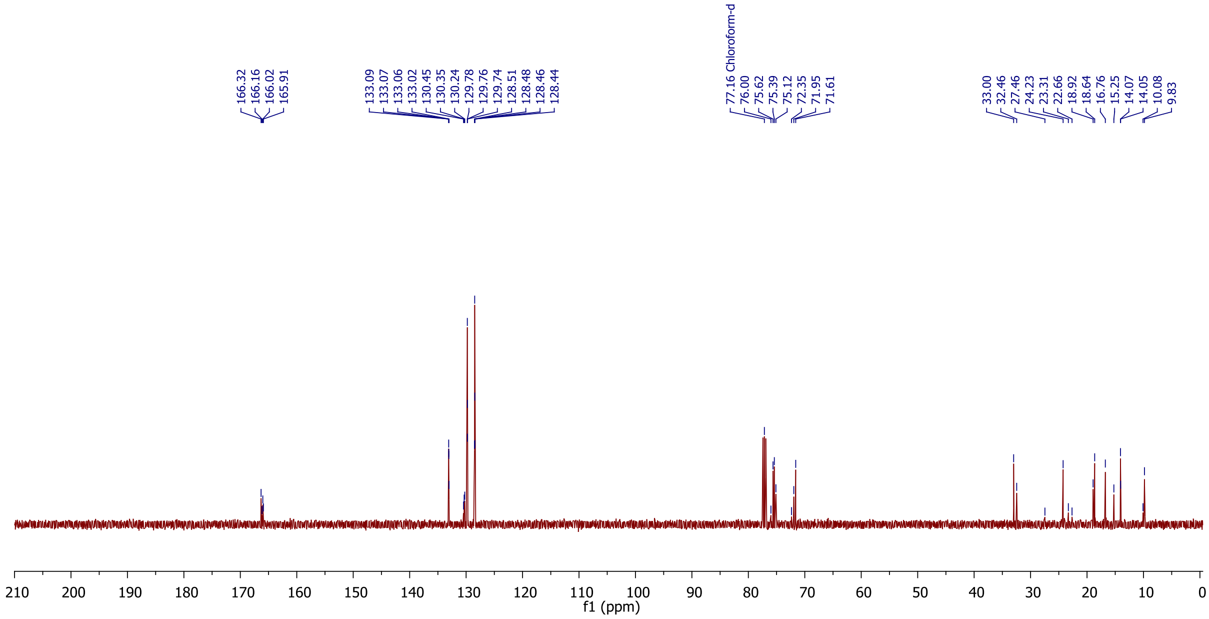

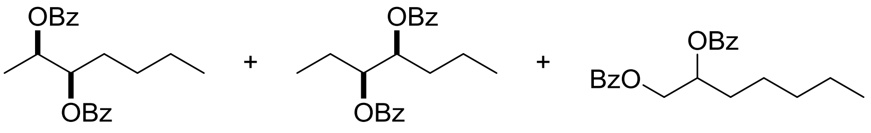

22a **22b** **22c**

Regioisomeric ratio (r.r.) = 5.5:2:1

^{1}H NMR spectrum of isomeric mixture of heptane-2,3-diyl dibenzoate (**22a**) with heptane-3,4-diyl dibenzoate (**22b**) and heptane-1,2-diyl dibenzoate (**22c**).

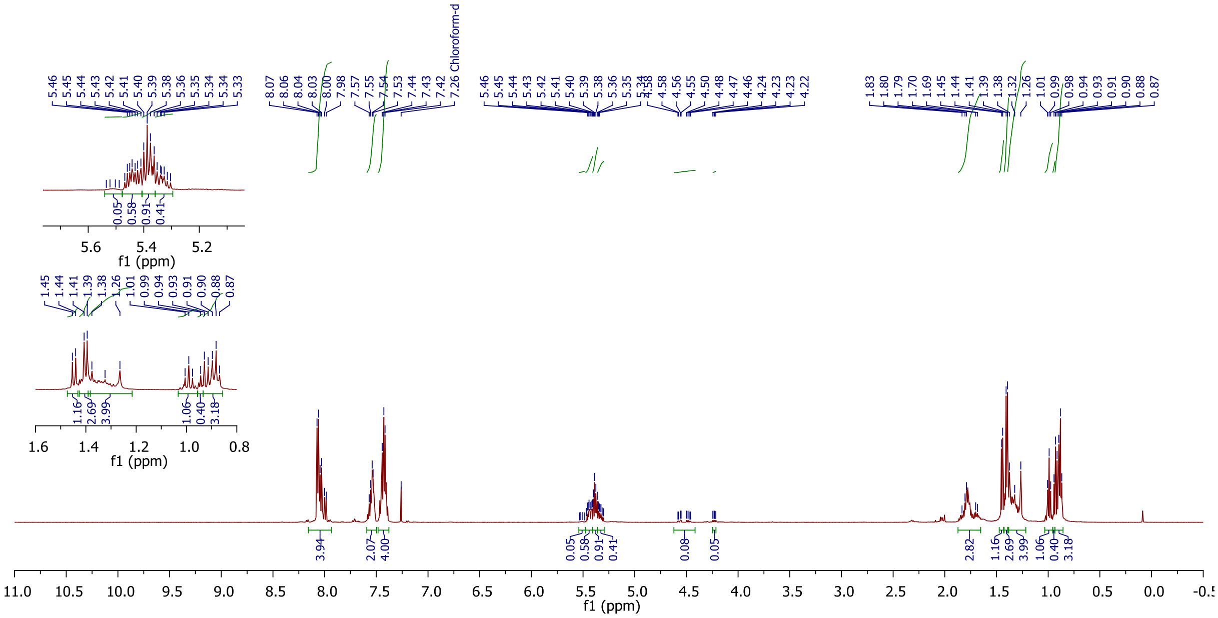

^{13}C NMR spectrum of isomeric mixture of heptane-2,3-diyl dibenzoate (**22a**) with heptane-3,4-diyl dibenzoate (**22b**) and heptane-1,2-diyl dibenzoate (**22c**).

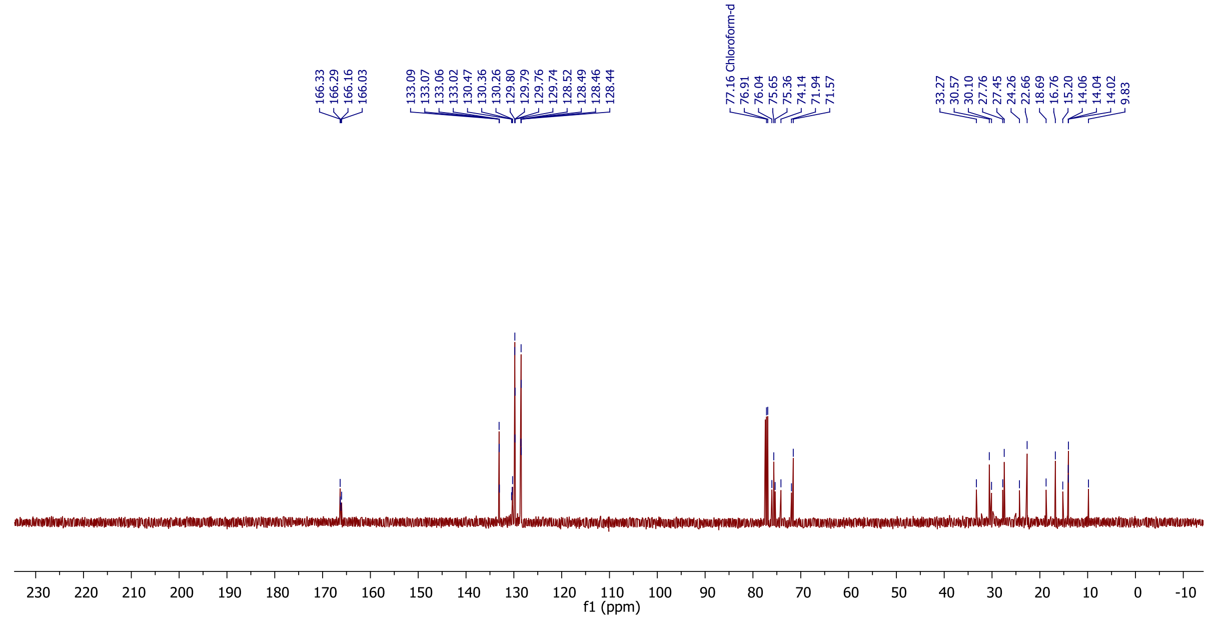

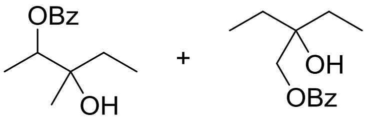

24a **24b**

Regioisomeric ratio (r.r.) = 7.7:1

^{1}H NMR spectrum of isomeric mixture of 3-hydroxy-3-methylpentan-2-yl benzoate (**24a**) with 2-ethyl-2-hydroxybutyl benzoate (**24b**).

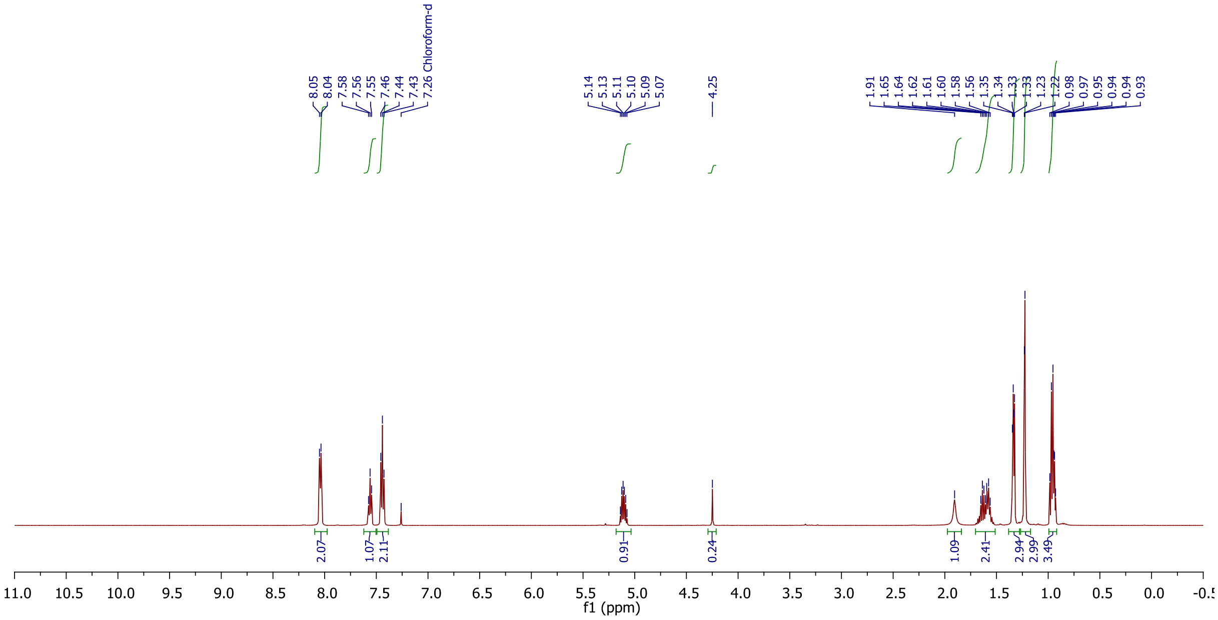

^{13}C NMR spectrum of isomeric mixture of 3-hydroxy-3-methylpentan-2-yl benzoate (**24a**) with 2-ethyl-2-hydroxybutyl benzoate (**24b**).

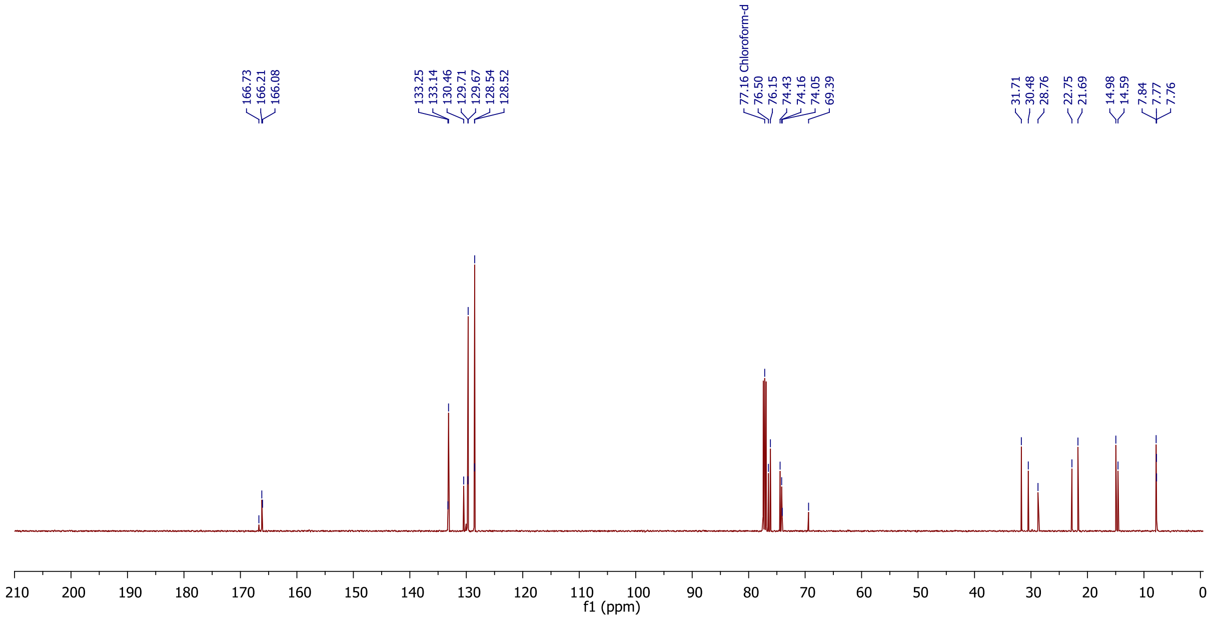

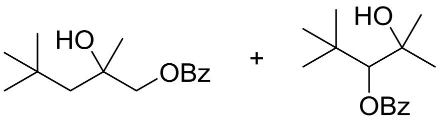

26a **26b**

Regioisomeric ratio (r.r.) = 8:1

^{1}H NMR spectrum of isomeric mixture of 2-hydroxy-2,4,4-trimethylpentan-3-yl benzoate (**26a**) with 2-hydroxy-2,4,4-trimethylpentyl benzoate (**26b**).

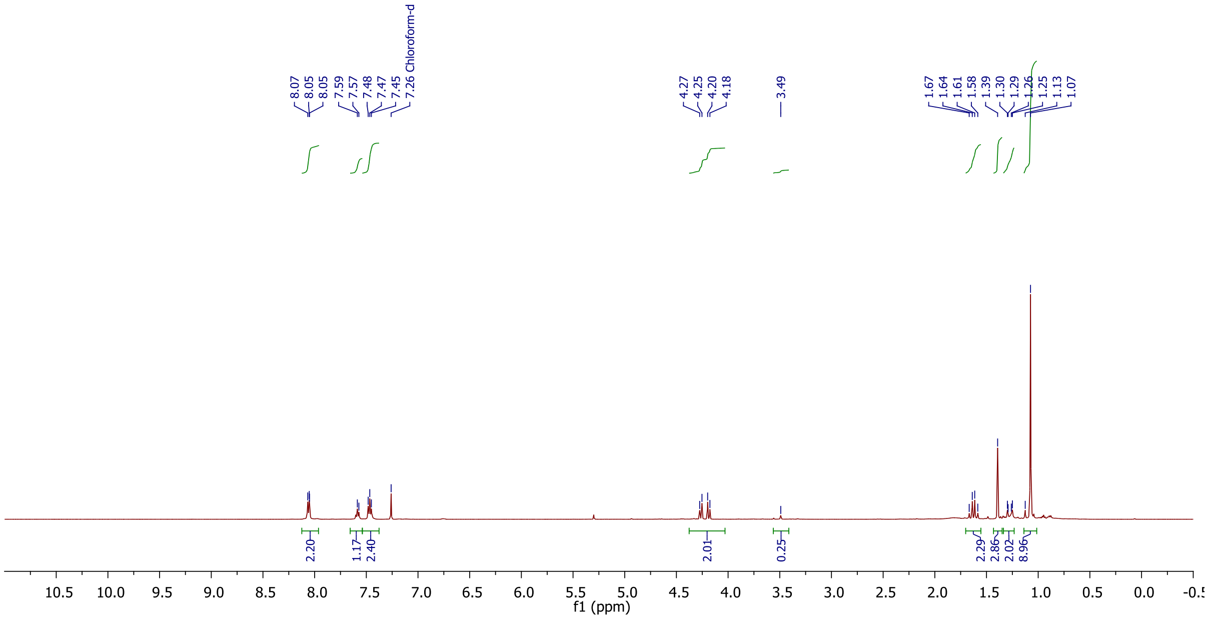

^{13}C NMR spectrum of isomeric mixture of 2-hydroxy-2,4,4-trimethylpentan-3-yl benzoate (**26a**) with 2-hydroxy-2,4,4-trimethylpentyl benzoate (**26b**).

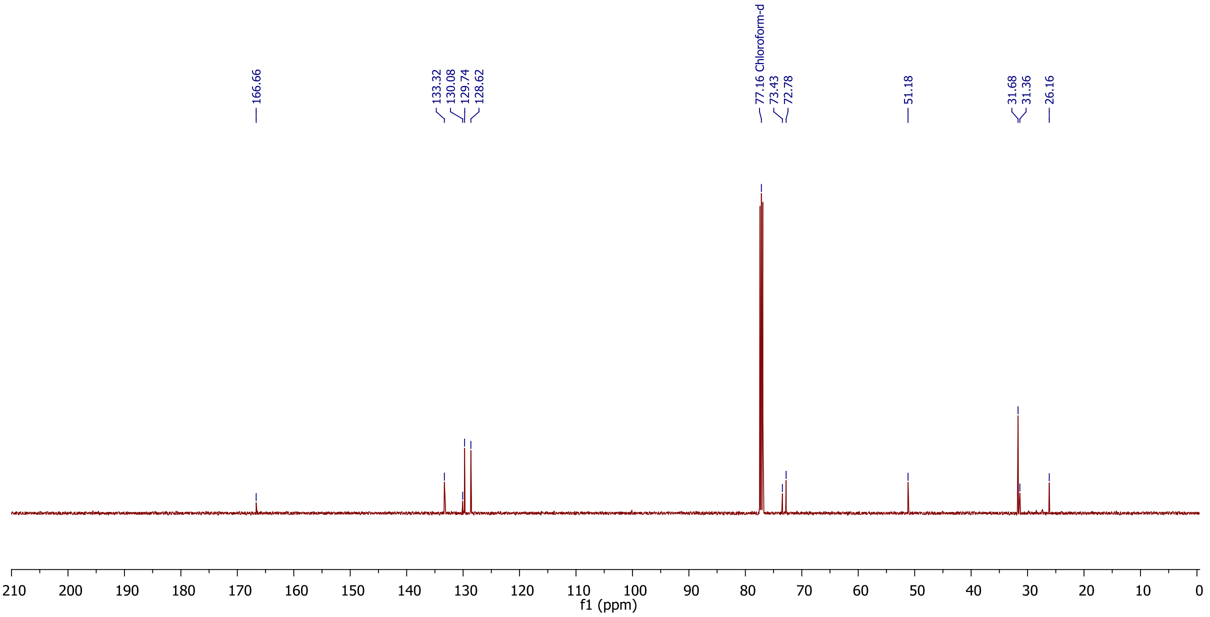

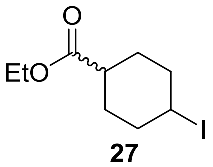

27

^{1}H NMR spectrum of ethyl 4-iodocyclohexane-1-carboxylate (**27**).

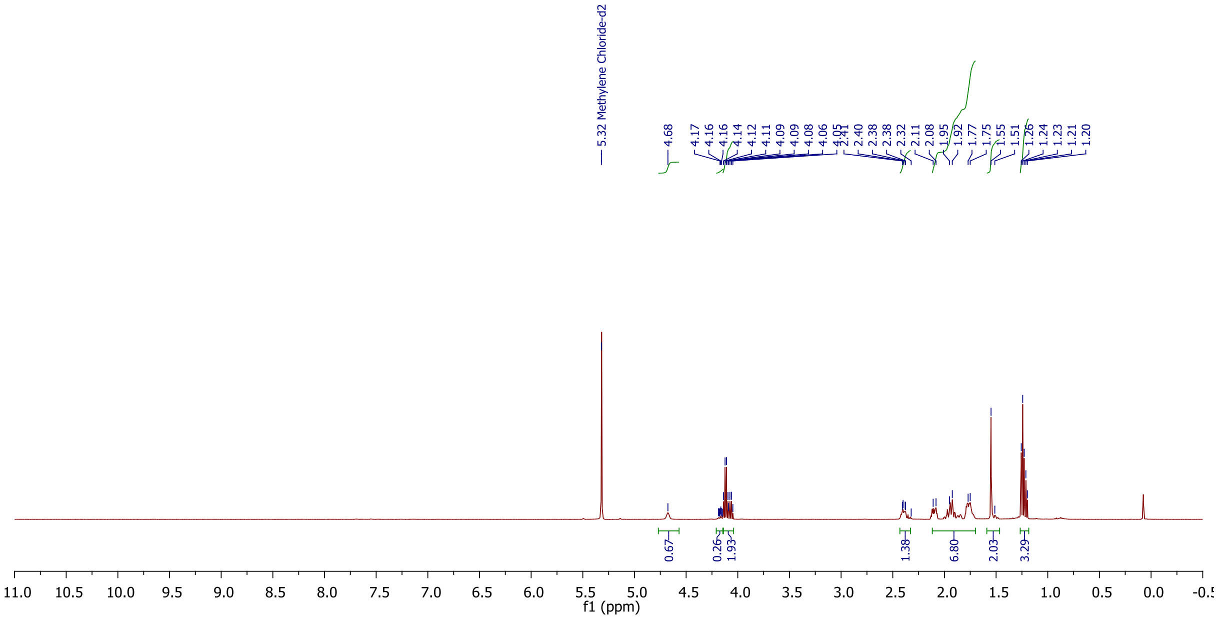

^{13}C NMR spectrum of ethyl 4-iodocyclohexane-1-carboxylate (**27**).

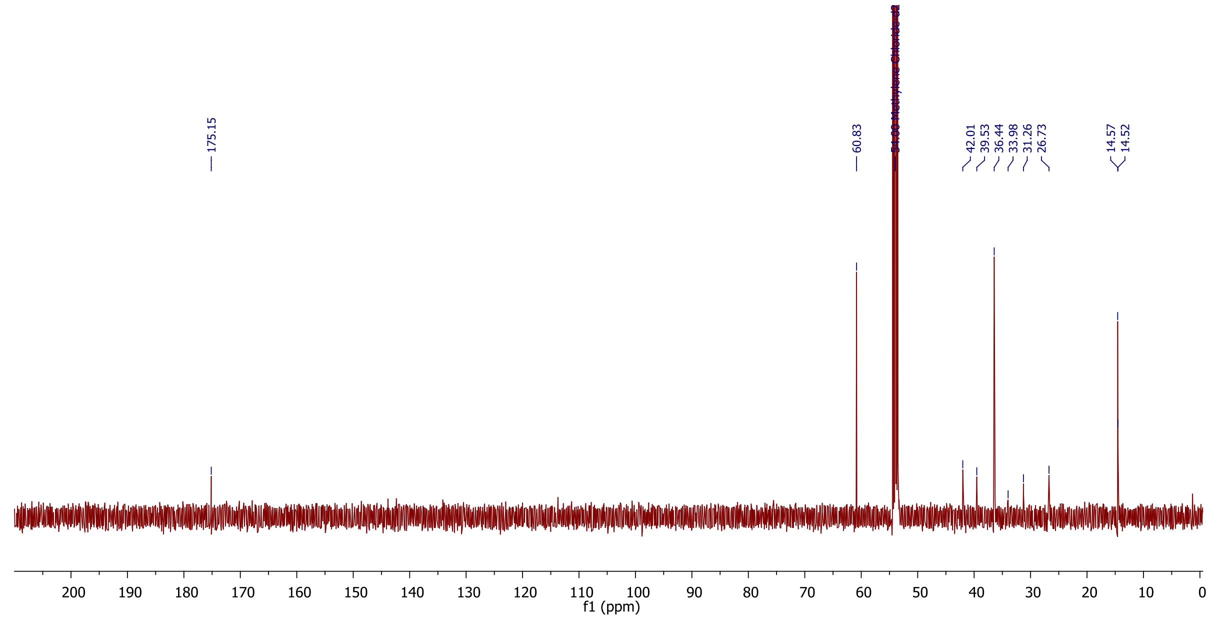

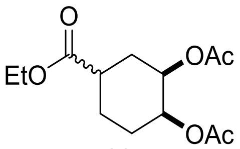

28

^{1}H NMR spectrum of 4-(ethoxycarbonyl)cyclohexane-1,2-diyl diacetate (**28**).

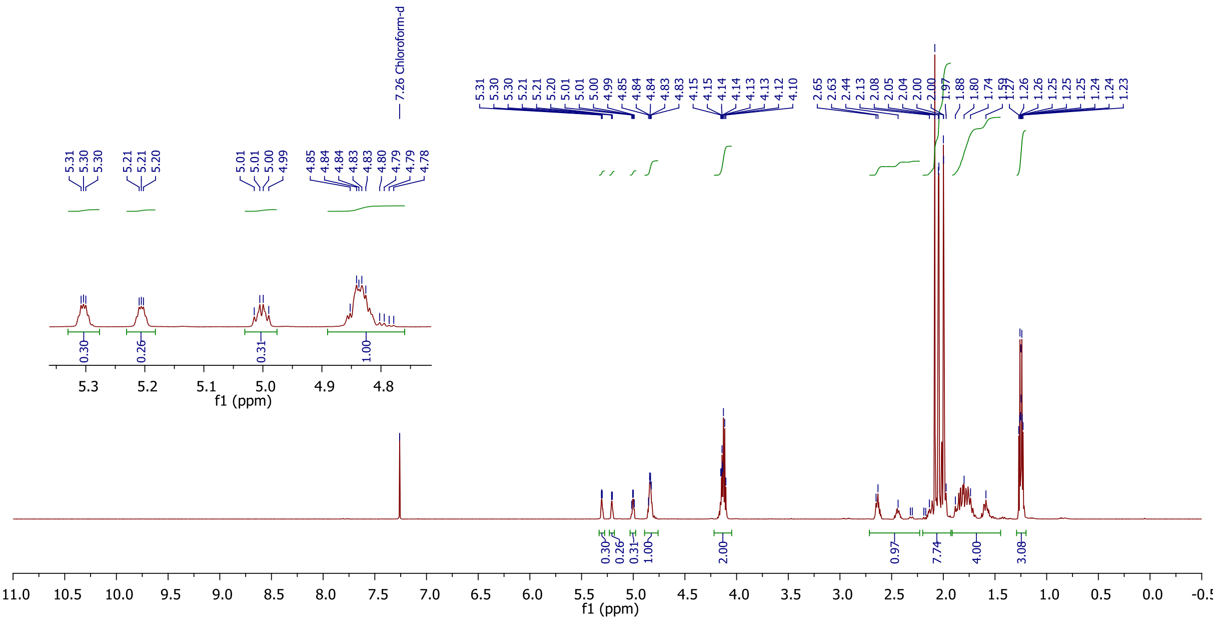

^{13}C NMR spectrum of 4-(ethoxycarbonyl)cyclohexane-1,2-diyl diacetate (**28**).

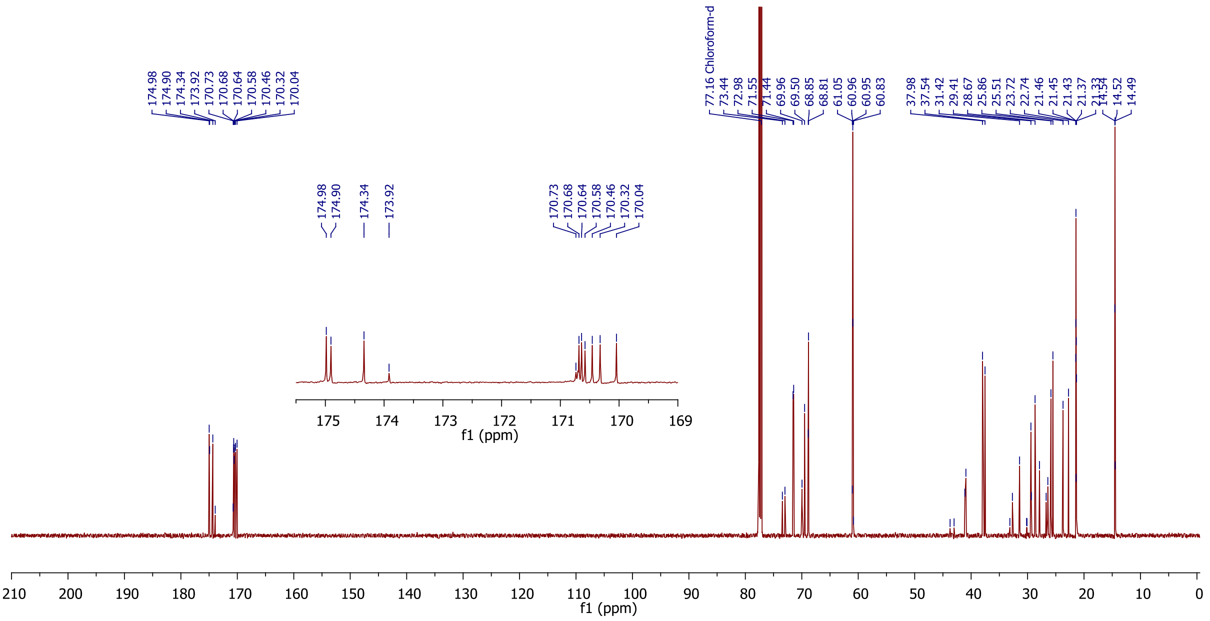

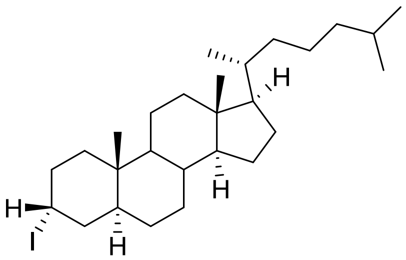

29

^{1}H NMR spectrum of 3α-iodo-5 α-cholestane (**29**).

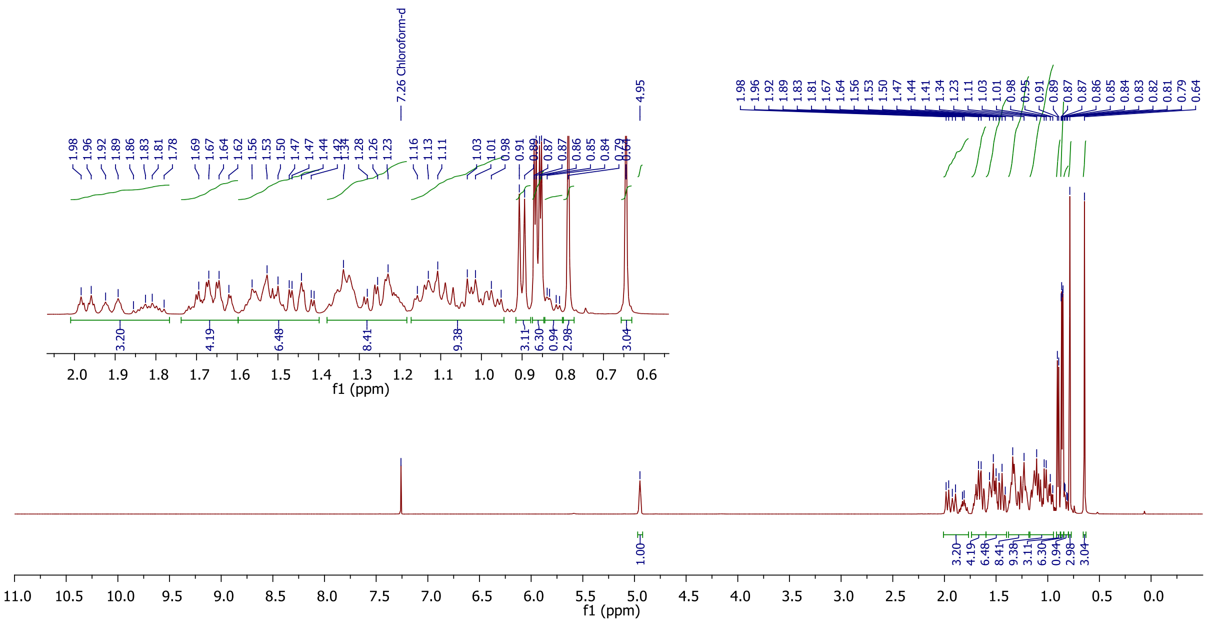

^{13}C NMR spectrum of 3α-iodo-5 α-cholestane (**29**).

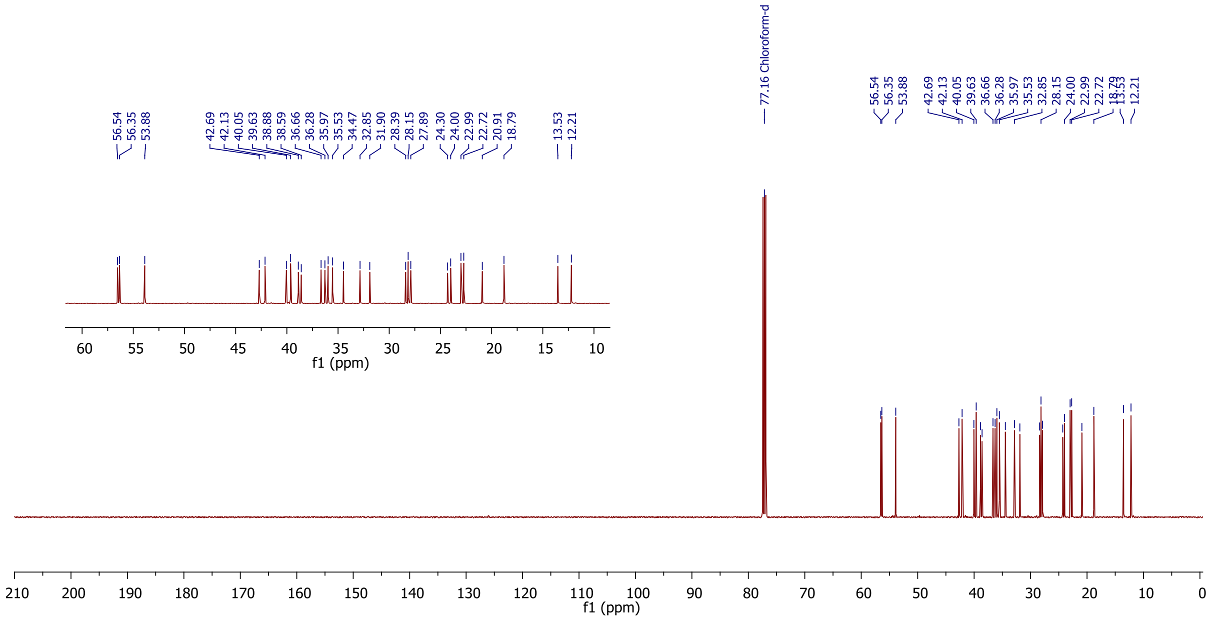

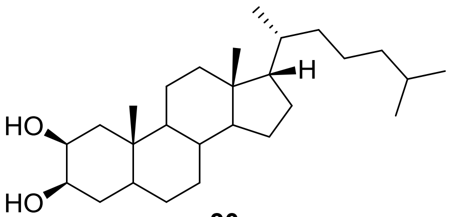

30

^{1}H NMR spectrum of spectrum of cholestane-2β,3β-diol (**30**).

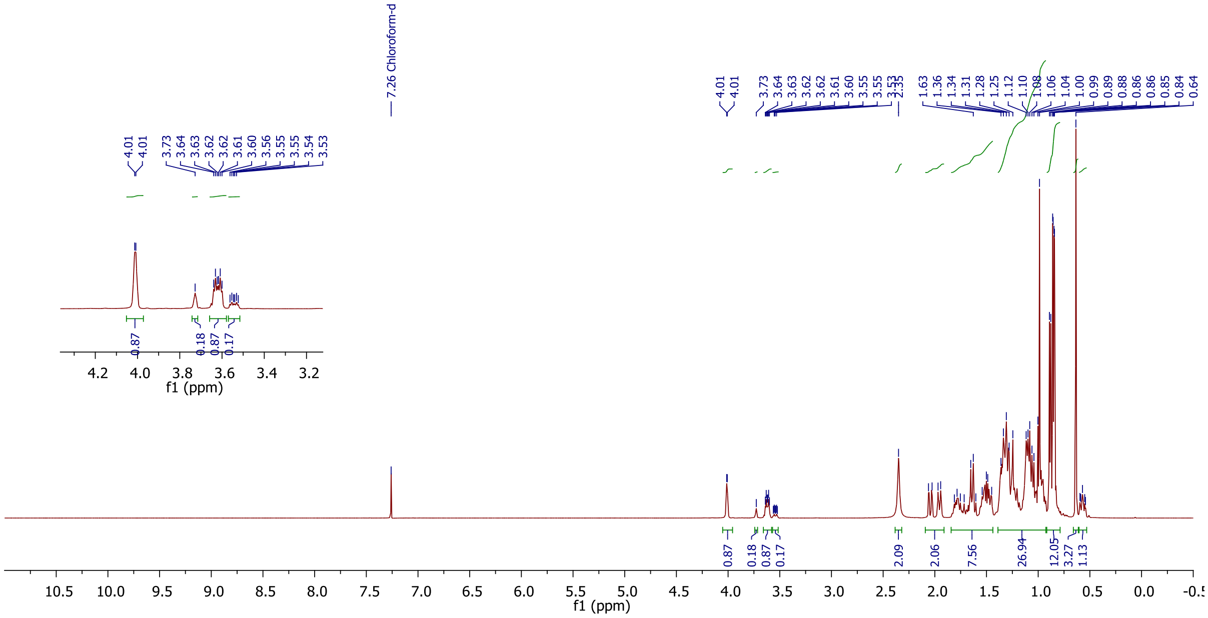

^{13}C NMR spectrum of cholestane-2β,3β-diol (**30**).

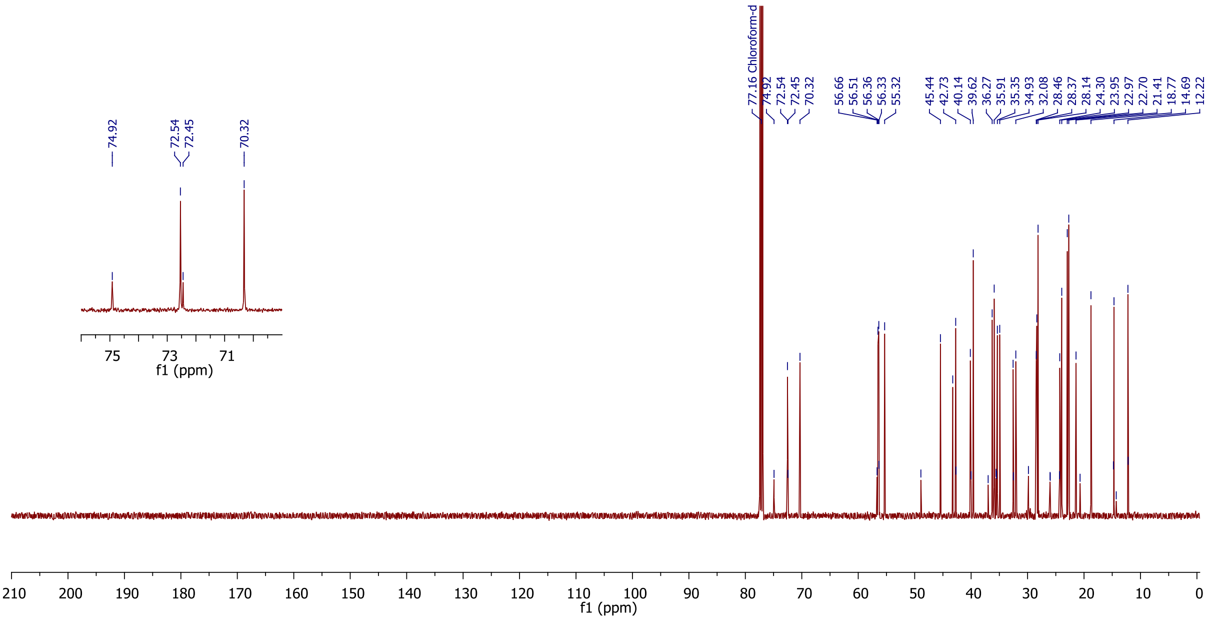

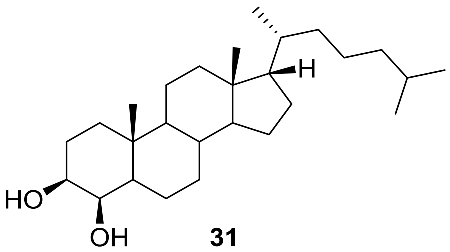

31

^{1}H NMR spectrum of cholestane-3β,4β-diol (**31**).

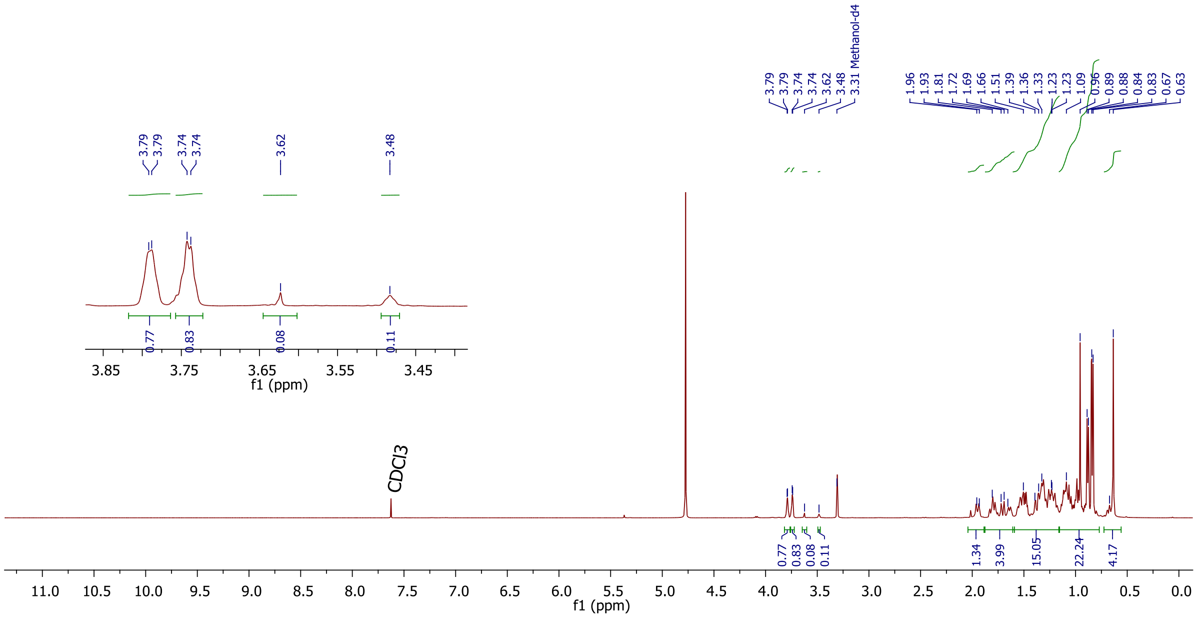

^{13}C NMR spectrum of cholestane-3β,4β-diol (**31**).

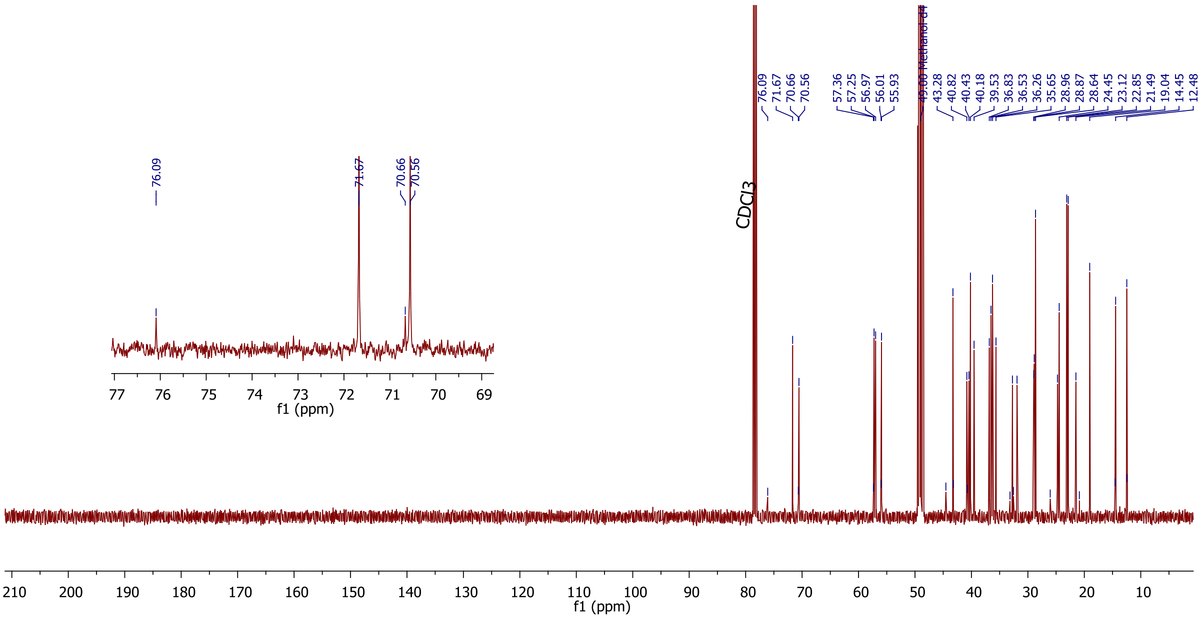

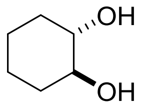

32

^{1}H NMR spectrum of cyclohexane-1,2-*trans*-diol (**32**).

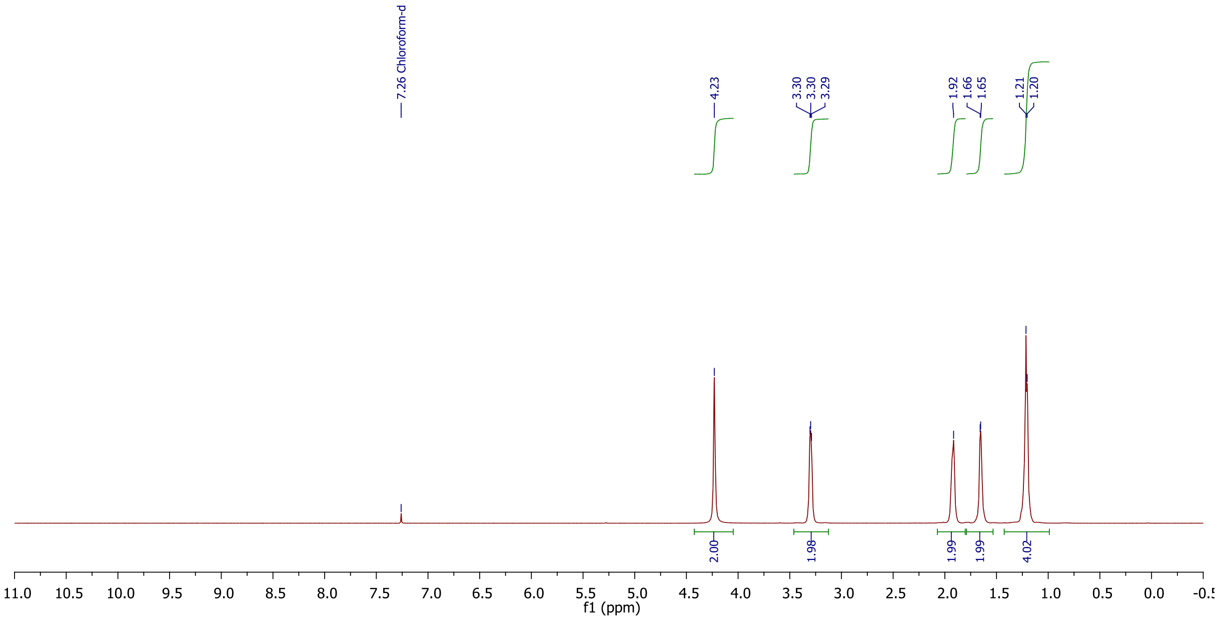

^{13}C NMR spectrum of cyclohexane-1,2-*trans*-diol (**32**).

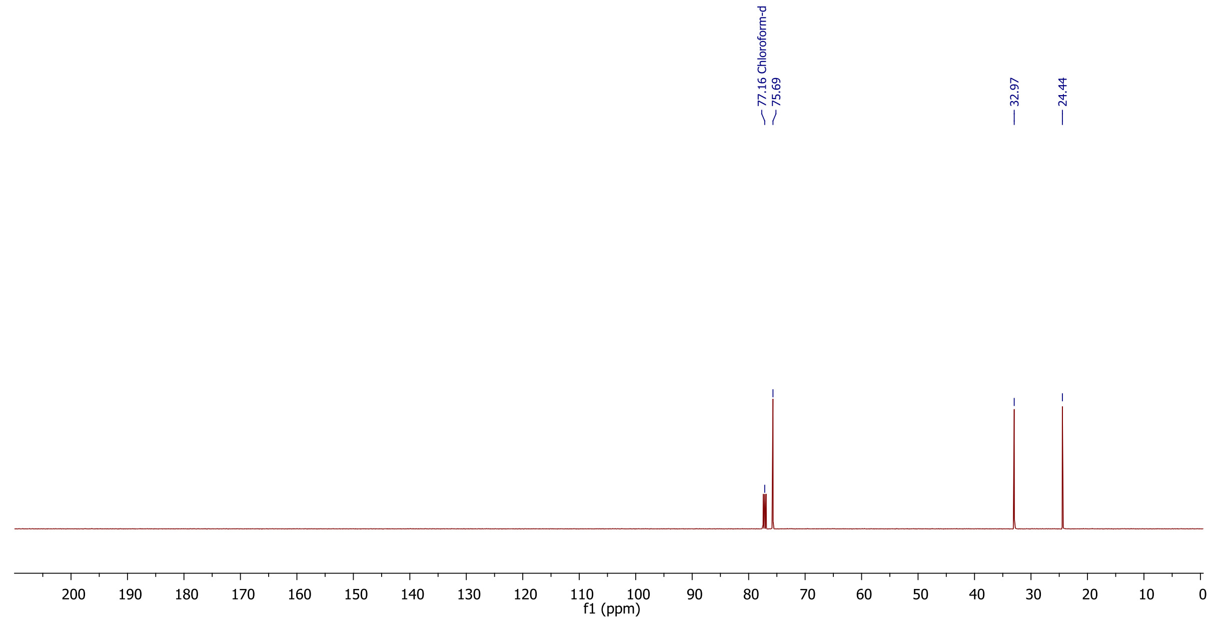

References

1) S. Nagayama, M. Endo, S. Kobayashi *J. Org. Chem.*, **1998**, *63*, 6094-6095.

2) E. Jahn, J. Smrček, R. Pohl, I. Císařová, P. G. Jones, U. Jahn, *Eur. J. Org. Chem.* **2015,** 7785–7798.

3) L. Emmanuvel, T. M. A. Shaikh, A. Sudalai, *Org. Lett.*, **2005,** *7,* 5071–5074.

4) X.-L. Geng, J. Wang, G.-X. Li, P. Chen, S. F. Tian, J. Qu, *J. Org. Chem.* **2008,** *73*, 8558–8562.

5) A. W. McDonagh, M. F. Mahon, P. V. Murphy, *Org. Lett.* **2016**, *18*, 552–555.

6) M. M. Cruz Silva, Sergio Riva, M. L- Sá e Melo, *Tetrahedron*, **2005,** *61,* 3065-3073.

7) A. W. Schmidt, T. Doert, S. Goutal, M. Gruner, F. Mende, T. V. Kurzchalia, H-J. Knölker Eur. J. Org. Chem. 2006, 3687–3706.
